# Sulfonated red and far-red rhodamines to visualize SNAP- and Halo-tagged cell surface proteins†

**DOI:** 10.1039/d1ob02216d

**Published:** 2022-02-21

**Authors:** Ramona Birke, Julia Ast, Dorien A. Roosen, Joon Lee, Kilian Roßmann, Christiane Huhn, Bettina Mathes, Michael Lisurek, David Bushiri, Han Sun, Ben Jones, Martin Lehmann, Joshua Levitz, Volker Haucke, David J. Hodson, Johannes Broichhagen

**Affiliations:** Leibniz-Forschungsinstitut für Molekulare Pharmakologie Berlin Germany broichhagen@fmp-berlin.de; Institute of Metabolism and Systems Research (IMSR), University of Birmingham Birmingham B15 2TT UK d.hodson@bham.ac.uk; Centre for Endocrinology, Diabetes and Metabolism, Birmingham Health Partners Birmingham B15 2TT UK; Centre of Membrane Proteins and Receptors (COMPARE), University of Birmingham Birmingham UK; Department of Molecular Pharmacology and Cell Biology, Leibniz-Forschungsinstitut für Molekulare Pharmakologie Berlin Germany; Department of Biochemistry, Weill Cornell Medicine New York NY 10065 USA; Department of Chemical Biology, Max Planck Institute for Medical Research Heidelberg Germany; Structural Chemistry and Computational Biophysics, Leibniz-Forschungsinstitut für Molekulare Pharmakologie Berlin Germany; Section of Endocrinology and Investigative Medicine, Imperial College London London W12 0NN UK; Oxford Centre for Diabetes, Endocrinology and Metabolism, Radcliffe Department of Medicine, University of Oxford Churchill Hospital Oxford UK

## Abstract

The (in)ability to permeate membranes is a key feature of chemical biology probes that defines their suitability for specific applications. Here we report sulfonated rhodamines that endow xanthene dyes with cellular impermeability for analysis of surface proteins. We fuse charged sulfonates to red and far-red dyes to obtain Sulfo549 and Sulfo646, respectively, and further link these to benzylguanine and choloralkane substrates for SNAP-tag and Halo-tag labelling. Sulfonated rhodamine-conjugated fluorophores maintain desirable photophysical properties, such as brightness and photostability. While transfected cells with a nuclear localized SNAP-tag remain unlabelled, extracellular exposed tags can be cleanly visualized. By multiplexing with a permeable rhodamine, we are able to differentiate extra- and intracellular SNAP- and Halo-tags, including those installed on the glucagon-like peptide-1 receptor, a prototypical class B G protein-coupled receptor. Sulfo549 and Sulfo646 also labelled transfected neurons derived from induced pluripotent stem cells (iPSCs), allowing STED nanoscopy of the axonal membrane. Together, this work provides a new avenue for rendering dyes impermeable for exclusive extracellular visualization *via* self-labelling protein tags. We anticipate that Sulfo549, Sulfo646 and their congeners will be useful for a number of cell biology applications where labelling of intracellular sites interferes with accurate surface protein analysis.

## Abstract 2

Sulfonated rhodamine dyes allow SNAP- and Halo-tag labelling of cell surface protein fusions. A far-red version can be used for STED nanoscopy.

## Graphical abstract

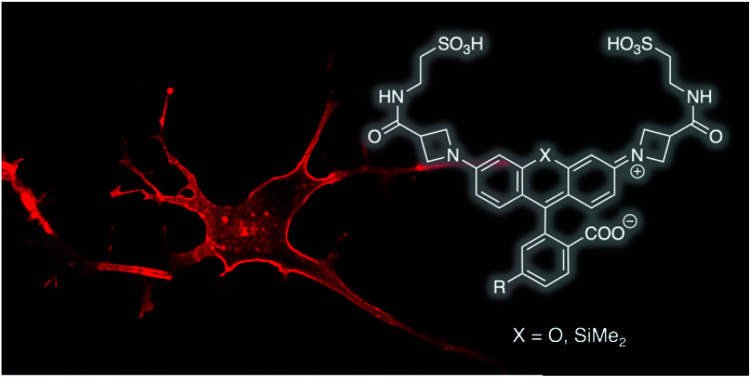

## Introduction

**Fig. 1:**
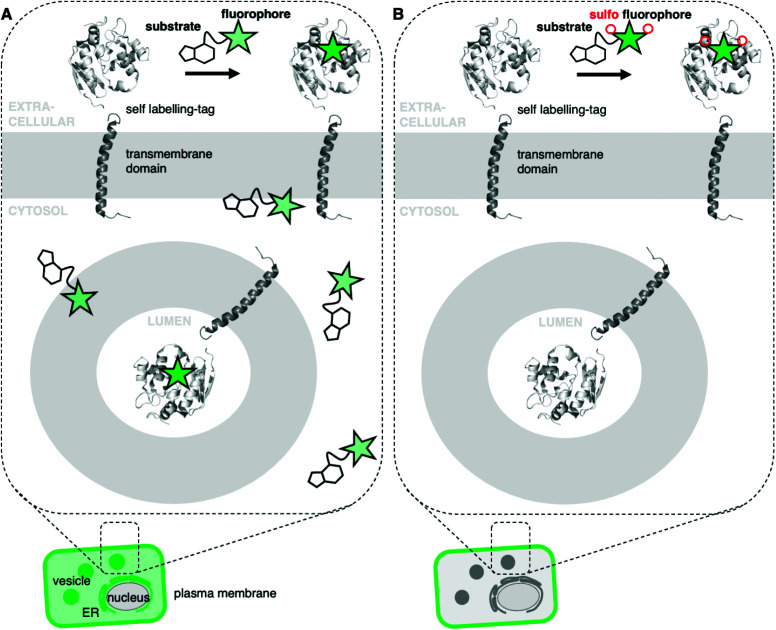
Permeable and impermeable fluorophores for protein labelling. (A) A self-labelling tagged transmembrane protein is localized at the cell surface in addition to different intracellular, membrane-enclosed compartments (*e.g.* vesicles, ER) and is visualized by staining with a fluorescent dye. Unbound dyes lead to non-specific background signals. (B) Sulfonation prevents the dye from entering the cell and therefore cleanly labels surface proteins, isolating them for visualization/interrogation.

Fluorescent microscopy is often the method of choice to visualize and interrogate cell biology.^1,2^ Two major methods can be distinguished: the use of genetically-encoded fluorescent proteins or the use of small molecule fluorophores.^2^ The latter can be targeted by chemical fusion to a selective and tight small molecule binder, or by means of self-labelling protein tags.^3–5^ A plethora of fluorescent small molecules spanning different photophysical and chemical properties are available for microscopy.^6,7^ Desirable properties for such fluorophores are brightness, resistance to photobleaching, and cellular permeability.^8–12^ Depending on the imaging modality, other properties might also be advantageous, such as blinking or fluorogenicity. However, very few fluorescent dyes exist for exclusive SNAP- and Halo-tag labelling on cell surface proteins, such as transmembrane receptors. Within the repertoire of membrane-impermeable fluorophores, even fewer are suitable for stimulated emission depletion (STED) nanoscopy,^13^ since higher laser powers are required that may lead to photobleaching.^14^ While several methods for protein labelling exist,^15^ targeting robust, permeable dyes to extracellular SNAP- or Halo-tags may lead to non-specific background and/or labelling of intracellular protein populations, especially in live cell applications.^16^ This is the case for visualizing surface membrane receptor localization (Fig. 1A), since it remains difficult to distinguish surface and intracellular pools of receptors by means of fluorescent labelling.^17^ To restrict *a priori* fluorogenic dyes to the cell surface, we set out to synthesize rhodamines endowed with a cell impermeable sulfonate moiety. To achieve this, the rhodamine scaffold of fluorogenic and bright JaneliaFluor (JF) dyes^8^ was extended with a short sulfonate-containing linker to provide red and far-red colors (Sulfo549 and Sulfo646), which can be targeted to cell surface tags and receptors (Fig. 1B). The versatility of this approach is highlighted in live cell imaging with various tagged constructs including G protein-coupled receptors (GPCRs) in different live cell types such as human induced pluripotent stem cells (iPSCs)-derived neurons, and in fixed neurons by STED nanoscopy to super-resolve axonal membranes.

## Results

**Fig. 2:**
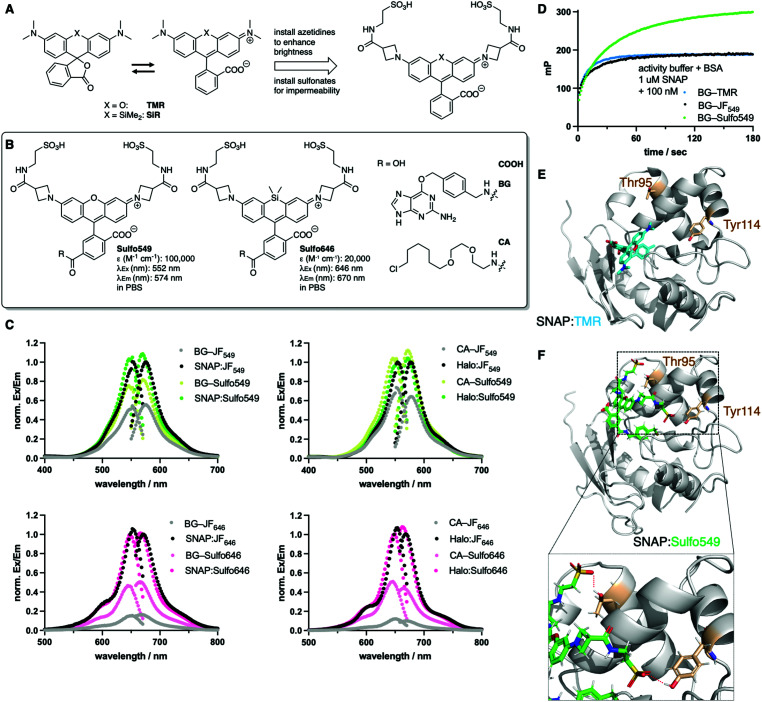
Design and properties of sulfonated rhodamines. (A) Rhodamines display fluorogenic behavior between a closed, non-fluorescent form, and an open, fluorescent isomer. Azetidine installation enhances brightness, and can be further derivatized with sulfonates. (B) Sulfo549 and Sulfo646 as red and far-red fluorophores can be linked to BG and CA substrates for SNAP- and Halo-tag labelling, respectively. (C) Normalized fluorescence excitation and emission spectra of non-sulfonated JF dyes and Sulfo dyes in solution and reacted with respective self-labelling tags. (D) Fluorescence polarization assay following the labelling reaction of SNAP. (E) Crystal structure of SNAP:TMR (pdb: 6y8p). (F) Best docking pose of SNAP:Sulfo549 reveals hydrogen bonding between the sulfonates and Thr95 and Tyr114 (red dashes in lower panel zoom).

We set out to synthesize impermeable dyes by the synthetic addition of charged sulfonates, which remain deprotonated and therefore render dyes not only cell impermeable, but may also increase their solubility in aqueous media. With our primary aims in mind, *i.e.* (i) impermeability, (ii) labelling of SNAP- and Halo-tags, and (iii) usage in STED nanoscopy, we decided to use xanthene dyes as a blueprint for our design. Xanthenes with a carboxylic acid in the 3-position on the lower phenyl ring are known to exist in two states, an open (fluorescent) and a closed (non-fluorescent) form (Fig. 2A) and are among the most stable towards photobleaching.^18^ The recently reported JaneliaFluor (JF) dyes are rhodamine-based fluorophores, which show higher brightness and fluorogenicity than their tetramethyl rhodamine congeners due to the installment of azetidines as nitrogen-containing moieties,^8^ suppressing non-radiative twisted intramolecular charge transfer (TICT) pathways.^19^ As such, we synthesized Sulfo549 and Sulfo646 congeners by introducing a carboxylate handle on the 3-position of the azetidine, which was further derivatized to a sulfonated head group *via* peptide coupling to taurine (Fig. 2A and Scheme S1†). A carboxylate in the 6-position served as a position to install *O*^6^-benzylguanine (BG) or a chloroalkane (CA) group, which act as substrates for the self-labelling SNAP- and Halo-tag, respectively, and thereby obtained four molecules displaying two colors and two labelling modalities (Fig. 2B and Scheme S1†). In a first set of experiments, we determined extinction coefficients for the free acids in PBS (*ε*(Sulfo549) = 100 000 M^−1^ cm^−1^ and *ε*(Sulfo646) = 20 000 M^−1^ cm^−1^) (Fig. 2B) while monitoring their stability *via* LCMS (ESI Fig. S1†), and further assessed the excitation and emission profiles of our dyes in their unbound (*i.e.* BG- and CA-linked) and bound (*i.e.* SNAP- and Halo-tag reacted) states (Fig. 2C). Fluorogenicity was reduced as expected when charges are added in close proximity to the dye, however, all dyes still showed high brightness (ESI Fig. S2 and S3†). Labelling was confirmed *in vitro* by incubation of Sulfo dyes with recombinant SNAP- and Halo-tag and subsequent mass spectrometry (see ESI†). Furthermore, we assessed kinetics of BG-Sulfo549 (*t*_1/2_ = 28.0 s) labelling on SNAP-tag *versus* BG-TMR (*t*_1/2_ = 8.9 s) and BG-JF_549_ (*t*_1/2_ = 15.3 s) by means of fluorescent polarization, and found a slight decrease in rate of labelling by a factor of ∼3.14 and ∼1.83, respectively (Fig. 2D). Still, full labelling of SNAP:Sulfo549 was achieved within minutes and, interestingly, with enhanced polarization, which led us to the hypothesis that the sulfonates might exhibit secondary interaction sites on the protein surface. Therefore, we performed a molecular docking study of Sulfo549 with SNAP protein using the program GOLD^20^ (version 2021.2.0, Cambridge Crystallographic Data Center). Compared to the X-ray crystallographic structure of TMR-bound SNAP-tag (pdb: 6y8p)^21^ (Fig. 2E) we indeed found a different dye geometry of Sulfo549 when covalently linked *via* its reactive cysteine145 (Fig. 2F). This can be further validated, since the sulfonates make hydrogen bond contacts with threonine95 and tyrosine114, thereby placing the ligand closer to the protein surface (for more details, see ESI Fig. S4†).

**Fig. 3:**
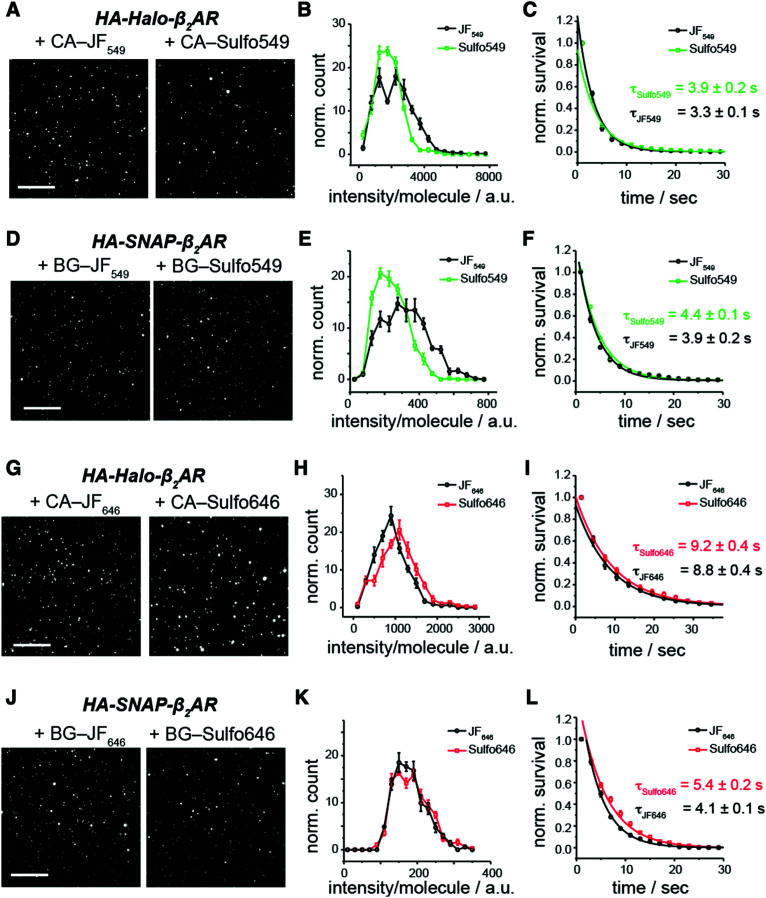
Single molecule intensity and photostability of sulfonated rhodamines. (A–L) Representative single molecule images (A, D, G and J) (scale bar = 10 μm), single molecule fluorescence intensity histograms (B, E, H and K) and survival plots as a measure of bleaching (C, F, I and L) for both parent JF and Sulfo dyes conjugated to SNAP- or Halo-tagged β_2_AR. Note that Halo constructs were tested with higher laser intensities than SNAP constructs (∼7 mW mm^−2^*versus* ∼1 mW mm^−2^) to facilitate sufficient bleaching on the 30 s time scale, indicating that all fluorophores are substantially more stable in the Halo context.

For further photophysical characterization we imaged immobilized single molecules of SNAP- or Halo-tagged membrane receptor (beta-2 adrenergic receptor; Halo-β_2_AR or SNAP-β_2_AR) labelled with either Sulfo dyes or their parent JF dyes (Fig. 3). This was performed in a single molecule pulldown (SiMPull) experiment, where immobilized receptors are linked to a coverslip and imaged by TIRF microscopy. In all cases, Sulfo dyes showed comparable fluorescence intensity and stability, indicating that installing a sulfonate did not impair the optimal properties of these fluorophores.

**Fig. 4:**
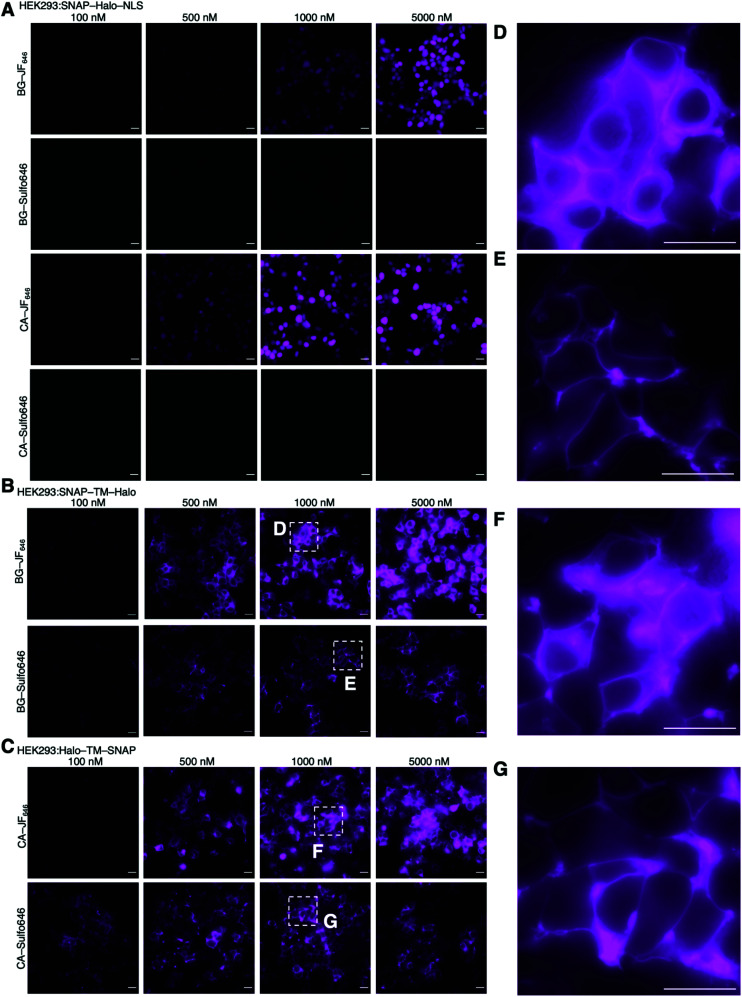
Titration of dyes in SNAP-Halo-NLS, SNAP-TM-Halo and Halo-TM-SNAP transfected HEK293 cells. (A) SNAP-Halo-NLS transfected HEK293 cells were treated with 100, 500, 1000 and 5000 nM of BG-JF_646_, BG-Sulfo646, CA-JF_646_ or CA-Sulfo646. (B) SNAP-TM-Halo transfected HEK293 cells were treated with 100, 500, 1000 and 5000 nM of BG-JF_646_ or BG-Sulfo646. (C) Halo-TM-SNAP transfected HEK293 cells were treated with 100, 500, 1000 and 5000 nM of CA-JF_646_ or CA-Sulfo646. (D, E, F and G) Zoom-ins from (B) and (C) highlighting surface staining for Sulfo646. NLS: nuclear localization signal; TM: transmembrane domain. Scale bar = 20 μm.

**Fig. 5:**
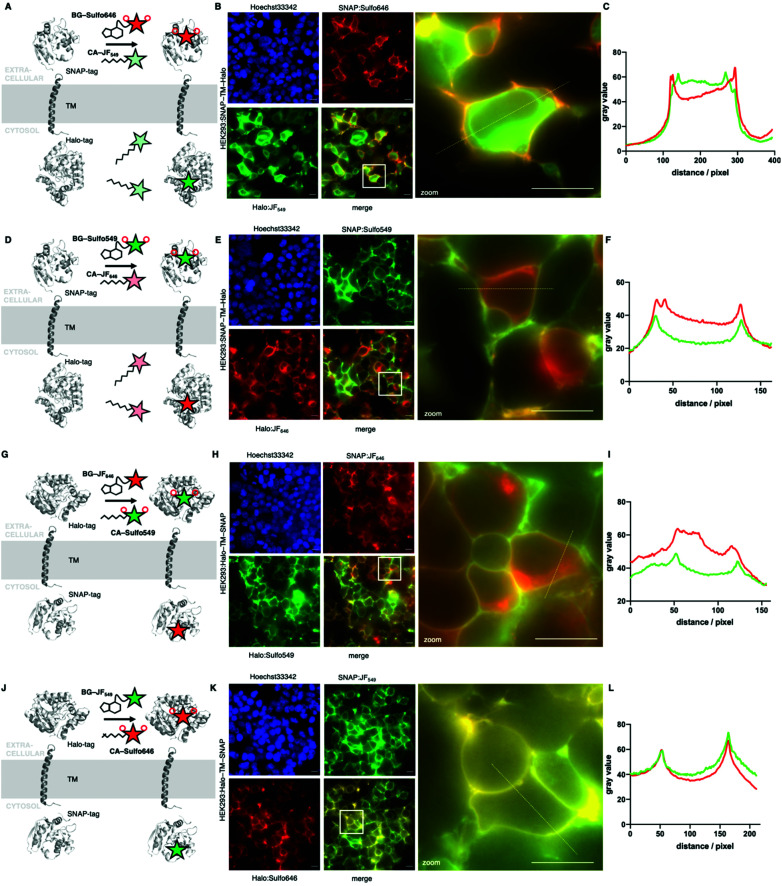
Widefield fluorescent imaging of live HEK293 cells transfected with SNAP-TM-Halo and Halo-TM-SNAP constructs. (A) Logic of labelling with BG-Sulfo646 and CA-JF_549_ leading to extra- and intracellular staining. (B) Widefield imaging of live, transfected, and labelled HEK293T cells; insert shows zoom-in. (C) Line plot intensity profile reveals membrane staining for impermeable Sulfo646 and intracellular labelling for JF_549_. (D–F) As for A, B, C, but with BG-Sulfo549 and CA-JF_646_. (G) Logic of labelling with CA-Sulfo549 and BG-JF_646_ leading to extra- and intracellular staining. (H) Widefield imaging of live, transfected, and labelled HEK293T cells, insert shows zoom-in. (I) Line plot intensity profile reveals membrane staining for impermeable Sulfo549 and intracellular labelling for JF_646_. (J–L) As for A, B, C, but with CA-Sulfo646 and BG-JF_549_. Scale bar = 20 μm.

Next, we wanted to test our molecules in a cellular setting for protein labelling in fluorescent microscopy. We expressed a dual SNAP-Halo-construct with a nuclear localization signal (NLS; SNAP-Halo-NLS^9^) in HEK293T cells, before titrating 100–5000 nM of permeable JF_646_ or its impermeable counterpart Sulfo646. Clear concentration-dependent nuclear signals were detected for JF dyes, but not for the Sulfo probes (Fig. 4A), indicating that incorporation of sulfonates indeed reduces or prevents cell permeability. To confirm that Sulfo dyes maintain the ability to label extracellular tags on the cell surface, we cloned two constructs containing: (i) an IgK trafficking signal for the plasma membrane; and (ii) a SNAP-tag and Halo-tag separated by a single pass transmembrane (TM) domain with the SNAP-tag or Halo-tag in either position (see ESI†). As such, the N-terminal tag (*i.e.* SNAP in SNAP-TM-Halo) should be labelled exclusively on the surface when using impermeable dyes, while a permeable dye would lead to additional staining from chimeric proteins residing inside the cell. Titration of 100–5000 nM of each dye led to increased non-specific signal when using JF_646_, whereas Sulfo646 labelled the cell surface, irrespective of the tag used (Fig. 4B and C). Furthermore, due to the installation of two orthogonal tags on the same construct, we were able to dual-color label with a red and far-red dye, testing for permeability and localization properties. Having established that 100 nM of substrate leads to sufficient labelling, HEK293T cells transfected with SNAP-TM-Halo (*i.e.* N-terminal SNAP and C-terminal Halo) were incubated with a combination of BG-Sulfo646/CA-JF_549_ each at 100 nM (Fig. 5A). Widefield fluorescence imaging revealed surface-localized staining for SNAP:Sulfo646 in combination with intracellular signals presumably originating from non-surface trafficked or nascent Halo:JF_549_ (Fig. 5B). This could be further resolved by a line profile through a transfected cell (Fig. 5C), which depicts plasma membrane and intracellular signals from the two colors. Using the same construct and settings, we then switched the dye colors for the respective tags. SNAP-TM-Halo-transfected HEK293T cells were labelled with BG-Sulfo549 and CA-JF_646_ (Fig. 5D), providing surface and intracellular staining for SNAP:Sulfo549 and Halo:JF_646_, respectively (Fig. 5E), as evidenced by line profile (Fig. 5F). Finally, experiments were repeated by switching the labelling tag localization and transfecting HEK293 cells with Halo-TM-SNAP construct, this time labelled with BG-JF_646_/CA-Sulfo549 (Fig. 5G). Widefield microscopy showed expected membrane-associated signals for Halo:Sulfo549 and intracellular signals for SNAP:JF_646_ (Fig. 5H and I). Using instead BG-JF_549_/CA-Sulfo646 (Fig. 5J), we were able to switch the localization to external Halo:Sulfo646 and internal SNAP:JF_549_ (Fig. 5K and L).

**Fig. 6:**
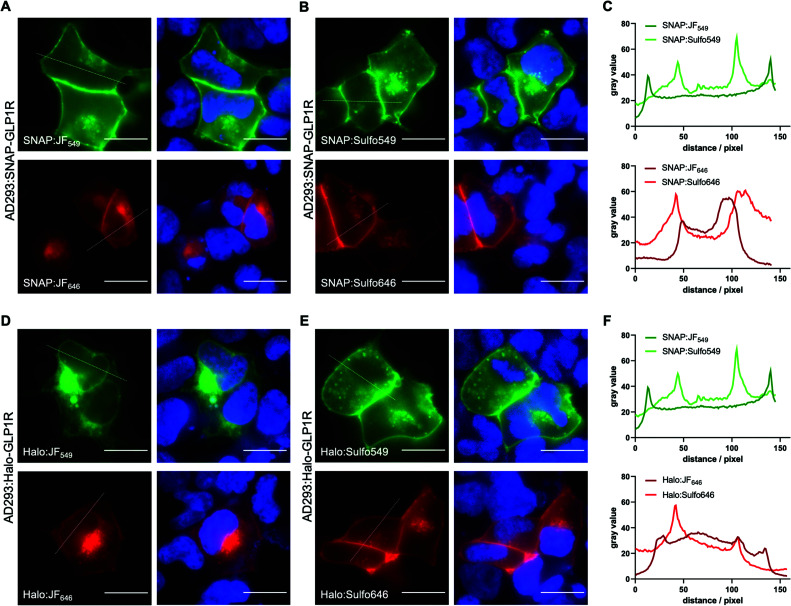
Live imaging of SNAP and Halo-tagged glucagon-like peptide-1 receptor (GLP1R). (A–C) Widefield imaging and line plot intensity profiles of AD293 cells expressing SNAP-GLP1R labelled with BG-JF_549_, BG-Sulfo549, BG-JF_646_, or BG-Sulfo646, and Hoechst33342 staining. (D–F) Same as for A–C, but AD293 cells expressing Halo-GLP1R and labelled with the corresponding CA dyes. Scale bar = 20 μm.

To test the utility of the Sulfo dyes for labelling cell surface receptors, we transfected AD293 cells with N-terminal SNAP- and Halo-tagged glucagon-like peptide-1 receptor (GLP1R), a class B GPCR and target for the incretin-mimetic class of anti-diabetic therapy^22^ (Fig. 6). Notably, a slight difference between BG-JF_549_ and BG-JF_646_ and their Sulfo derivatives was observed for SNAP labelling (Fig. 6A–C), which became more prominent for Halo labelling (Fig. 6D–F). We also transfected HEK293T cells with either N-terminally Halo- or SNAP-tagged beta-2 adrenergic receptor (Halo-β_2_AR or SNAP-β_2_AR) and labelled with either JF or Sulfo dyes. Consistent with studies of dual SNAP-Halo constructs (Fig. 5), clear intracellular labelling was only observed for JF dyes while Sulfo dyes displayed discrete fluorescence restricted to the outside of the cell, consistent with exclusive targeting to plasma membrane-localized receptors (ESI Fig. S5†).

**Fig. 7:**
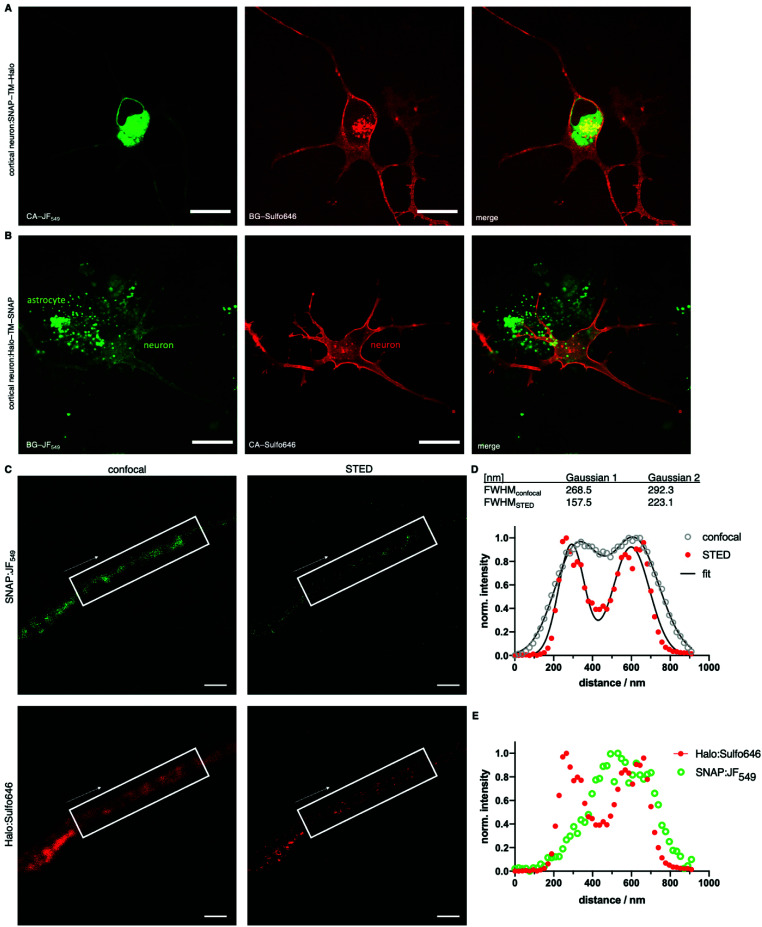
Confocal and STED imaging of live and fixed transfected human iPSC-derived cortical neurons. Neurons co-cultured with astrocytes were transfected with a SNAP-TM-Halo (A) or a Halo-TM-SNAP (B) construct and labelled with CA-JF_549_/BG-Sulfo646 (A) and BG-JF_549_/CA-Sulfo646 (B). Scale bar = 20 μm. (C) Cultures transfected with Halo-TM-SNAP from (A) were fixed and BG-JF_549_- and CA-Sulfo646-labelled axons imaged by confocal microscopy and STED nanoscopy. (D) Line profile along an axon showing improved resolution of Halo:Sulfo646 at the membranous margins in STED *versus* confocal imaging. (E) Halo:Sulfo646 allows visualization of surface signals, whereas SNAP:JF_549_ is largely confined to the intra-axonal compartment (STED signals). Scale bar = 1 μm.

Given the performance of the Sulfo dyes in heterologous cell lines, we sought to extend our studies to more complex cell populations where background signal can make accurate protein localization more difficult. Thus, glutamatergic neurons derived from human iPSCs co-cultured with murine primary astrocytes^23,24^ were transfected with SNAP-TM-Halo (*i.e.* N-terminal SNAP and C-terminal Halo) and Halo-TM-SNAP (*i.e.* N-terminal Halo and C-terminal SNAP) constructs. Labelling was performed with the respective impermeable far-red Sulfo646 and permeable red JF_549_, before live imaging by confocal microscopy. Clear plasma membrane staining was observed for the SNAP-TM-Halo construct labelled with BG-Sulfo646 (Fig. 7A), while CA-JF_549_ preferentially marked intracellular pools of SNAP-TM-Halo. When Halo-TM-SNAP was labelled with CA-Sulfo646 and BG-JF_549_ (Fig. 7B) bright neuronal labelling was only observed for Halo:CA-Sulfo646 and was restricted to the cell surface. By contrast, BG-JF_549_ non-specifically accumulated in co-cultured astrocytes. Thus, for both self-labelling tags, the Sulfo dyes demonstrated excellent performance for cell surface protein visualization in more complex cell types.

Lastly, we fixed Halo:Sulfo646 and SNAP:JF_549_ labelled neuronal cultures prior to analysis by STED nanoscopy (Fig. 7C). An accumulated line profile along an axon (white box) of the confocal and STED images revealed that Sulfo646 is amenable to nanoscopy (Fig. 7D). By two-Gaussian fitting, we obtained sharper full width half-maximal values for STED *versus* confocal (FWHM_confocal_ = 268.5 and 292.3 nm; FWHM_STED_ = 157.5 and 223.1 nm) microscopy. Consistent with our previous results, Halo:Sulfo646 generated a more pronounced signal along the axonal membrane, while SNAP:JF_549_ was observed in intra-axonal compartments (Fig. 7E).

## Discussion

The need for custom-tailored dyes is in high demand as the range of microscopy modalities, experimental techniques, experimental models and labelling strategies increases. Recent developments^9,25–28^ have focused mostly on boosting brightness, fluorescent lifetimes, chemical stability and/or fluorogenicity, the latter being a cause for cellular permeability. For the interrogation of cell surface proteins that are genetically fused to self-labelling protein tags (*e.g.* SNAP- and Halo-tag), however, cell impermeable dyes are desirable. Rendering dyes impermeable is usually achieved by introduction of sulfonates, which remain negatively charged in biological systems and are therefore not able to passively cross the plasma lipid bilayer. While many sulfonated dyes exist, such as Alexa488/568/647, or LD555/655,^29^ their application for enzyme self-labelling and STED nanoscopy has so far not reached the performance bar set by permeable dyes. A recent study, however, showed good performance of LD dyes for FRET measurements of dimer formation on SNAP-tagged GPCRs and in TIRF microscopy for single molecule FRET recovery after photobleaching.^30^ Rhodamine dyes have attracted some attention in recent years since the emergence of JaneliaFluor (JF) dyes, which rely on the exchange of azetidine groups for dimethylamines on various molecular scaffolds.^8^ Indeed, one impermeable version has been described, JF_635i_, which retains some of its fluorogenicity and has been used to observe Halo-tagged transferrin receptor recycling.^31^ Nevertheless, it has only been described as a Halo-tag substrate and has not been subjected to super-resolution STED nanoscopy. We aimed to expand this palette by using azetidine containing rhodamines for red and far-red imaging, which show minimal fluorogenicity and therefore maximal brightness. Accordingly, we synthesized Sulfo549 and Sulfo646 based on JF_549_ and JF_646_, each bearing two sulfonate groups, and further linked them to SNAP- and Halo-tag substrates BG and CA, respectively. Our probes complement a previous study where we reported a strategy to limit any dye to cell surface exposed SNAP-tags by altering the BG substrate to a sulfonate itself (termed SBG).^17^ With a simple chloride anion being the leaving group for the Halo-tag, introducing a charged sulfonate is not tolerated. Therefore, the impermeable characteristics need to instead be provided by the dye, for which we provide a solution herein. Indeed, with this toolset of impermeable dyes across the visible spectrum, this maneuver enables 3-color imaging of surface proteins, by using SNAP, CLIP and Halo-tags.^32^ In addition, we found a potential secondary binding site of SNAP:Sulfo549 by molecular docking, whereby both sulfonates form a hydrogen bond to the protein.

To test our approach, we performed single molecule benchmarking on SNAP- and Halo-β2AR to show that sulfonation is tolerated, and further cloned constructs for cellular transfections that bear a SNAP- and Halo-tag separated by a transmembrane domain, thereby placing the tags either side of the plasma membrane. These constructs allowed screening of permeability parameters in live HEK293 cells. Sulfo dyes performed well in these systems, where no background signals from intracellular spaces were detected. Encouraged by this, we next used SNAP- and Halo-tagged GLP1R to provide performance benchmarking in a more relevant cell surface signalling protein. Comparable to previous findings with the SNAP/Halo constructs, we obtained clean surface labelling for both colors and both protein tags, with more prominent effects for Halo, which is in line with *in vitro* measurements. Given that GLP1R labelling was performed at 37 °C, where constitutive activity of GPCRs can be increased, intracellular signal from Sulfo dyes can be observed, and we note that signal strength might also differ due to expression and endocytosis levels. Nonetheless, these results open up the possibility to perform SNAP-tag/Halo-tag dual color interrogation of post-endocytic protein trafficking^31,33^ and cell surface receptor ensembles,^34–36^ as we showed previously for the SNAP-tag alone.^17^

Finally, we extended this approach to more complex cell types by staining live somatodendritic and axonal compartments in iPSC-derived human glutamatergic neurons, co-cultured with astrocytes. By transfecting neurons with SNAP-TM-Halo or Halo-TM-SNAP, we aimed to benchmark far-red staining of the outer membrane with both tags, as this color was our choice for subsequent super-resolution imaging. As such, we observed surface labelling with both constructs and the use of the respective Sulfo646, with signals markedly brighter when bound to Halo. Of note, BG-JF_549_ accumulated non-specifically in astrocytes, while BG-JF_646_ labelled only neurons. While the reason for this observation is unknown, these results suggest caution when using BG-JF_549_ or its derivatives to label neuronal membranes when co-cultured with astrocytes. Self-labelling tags have been employed in “brainbow” labelling, where they offer more flexibility in terms of colors available, more straightforward applicability than antibodies, and better survival of the harsh clearing conditions with respect to fluorescent proteins.^37^ Indeed, we anticipate Sulfo dyes to be a favorable addition to such studies and their performance in whole tissues,^38,39^ and with this observed trend in mind, we chose to continue with Halo:Sulfo646 in our preparations. For this reason, we fixed the astrocyte/neuron co-culture and tested SNAP:JF_549_ and Halo:Sulfo646 for STED nanoscopy. As expected, JF_549_ was not amenable to the depletion laser. However, Sulfo646 was able to improve full width half-maximal values when a broad line plot was applied along an axon subjected to STED imaging. In addition, the resolution (*i.e.* the distance of the two maxima) of the membranes was ∼300 nm in both confocal and STED, consistent with prior studies.^40,41^

## Summary

We have designed and synthesized sulfonated fluorescent rhodamine dyes (Sulfo549 and Sulfo646) that are based on the JaneliaFluor scaffolds to obtain bright and impermeable dyes in the red and far-red ranges. By linking these dyes to substrates recognized by the SNAP and Halo-tag, we were able to achieve exclusive cell surface labelling in HEK293/AD293 cells and in human iPSC-derived neurons by means of widefield and confocal microscopy. Lastly, we employed STED nanoscopy on Sulfo646-labelled iPSC-derived neurons, showcasing the ability of the dyes to resolve axonal membranes. We anticipate that these and other sulfonated rhodamines will be useful for visualizing cell surface proteins using a range of imaging approaches spanning widefield through confocal through super-resolution.

## Methods

### Chemistry, cloning and *in vitro* protein labelling

Chemical schemes, synthetic protocols, protein labelling *in vitro*, and characterization can be found in the ESI.† Plasmids were cloned using a Gibson assembly cloning Kit (NEB), primers were designed using the NEBuilder assembly tool. Plasmids were isolated using a mini prep kit (Thermo Fisher). DNA concentration was measured on a NanoDrop (Thermo Fisher) and verified by Sanger sequencing (see ESI†).

### Stability studies

Sulfo549-6-COOH and Sulfo646-6-COOH were dissolved in DMSO to obtain a 1 mM stock solution, which was further diluted into PBS to yield a 20 μM solution that was kept at room temperature. LCMS was run immediately and at appropriate time points up to 24 hours, while observing no change in the chromatogram at the respective absorbance maxima.

### Docking

The complete structure of SNAP was generated using a local installation of AlphaFold v2.0.0. The interactions between the ligand Sulfo549 and SNAP were modelled using the docking program GOLD (version 2021.2.0, Cambridge Crystallographic Data Center)^20^ integrated in the software package DiscoveryStudio (BIOVIA), where ChemPLP scoring function was employed.^42^ Here we selected the binding site manually according to the binding site of SNAP and TMR in its X-ray structure (pdb code: 6Y8P)^21^ and applied a covalent restraint on the thioether bond between the Ligand Sulfo549 and Cys145. Eight out of ten best docking poses are rather convergent. The best pose is shown in Fig. 2F.

### *In vitro* fluorescence spectroscopy

Purified SNAP_f_ and Halo was obtained as previously described.^26^ Labelling dyes were dissolved in DMSO to a concentration of 1 mM and diluted in activity buffer (containing: 50 mM NaCl, 50 mM HEPES, pH = 7.3 + 4 μg mL^−1^ BSA) to 500 nM. Protein was diluted in activity buffer to a concentration of 2 μM. 100 μL of each protein and labelling agent were combined in each well in a black flat bottom 96-well plate and allowed to incubate at room temperature for 30 min, before fluorescence spectra were acquired on a TECAN infinite 2000Pro plate reader. Experiments were run in quadruplicate and plotted in GraphPad Prism 8.

Kinetic measurements were performed on a TECAN GENios Pro plate reader by means of fluorescence polarization. Stocks of SNAP_f_ (2 μM) and substrates (200 nM) were prepared in activity buffer (containing in mM: NaCl 50, HEPES 50, pH 7.3) with additional 10 μg mL^−1^ BSA. SNAP_f_ and substrates were mixed (100 μL each) in a Greiner black flat bottom 96-well plate. Mixing was performed *via* a built-in injector on a TECAN GENios Pro. Fluorescence polarization reading was started immediately (*λ*_Ex_ = 535 ± 25 nm; *λ*_Em_ = 590 ± 35 nm; 10 flashes; 40 μs integration time). Experiments were run in quadruplicates and raw polarization values were one-phase decay fitted in GraphPad Prism 8.

### Protein mass spectrometry

Labelling substrates were dissolved in DMSO to a concentration of 1 mM and diluted in activity buffer (containing: 50 mM NaCl, 50 mM HEPES, pH = 7.3 + 4 μg mL^−1^ BSA) to 20 μM. Protein was diluted in activity buffer to a concentration of 2 μM. 25 μL of each protein and labelling agent were combined in a mass spec vial and allowed to incubate at room temperature for 1 h, before full protein mass was acquired. For non-labelling control, 25 μL of activity buffer was mixed with 25 μL of protein.

### Single molecule pulldown (SiMPull)

For SiMPull, lysates were prepared from fluorescently labelled cells and HA-tagged SNAP-β_2_AR and Halo-β_2_AR constructs were immobilized in 0.1% DDM using a biotinylated anti-HA antibody as previously described.^17,43^ Single molecule movies were recorded as described previously.^17,35,43^ Laser lines of 561 nm or 640 nm were used to excite Sulfo549 (or JF_549_) or Sulfo646 (or JF_646_), respectively, and molecules were visualized with appropriate emission filters of 595/50BP (Chroma Technology) for Sulfo549 (or JF_549_) and 655LP (Chroma Technology) for Sulfo646 (or JF_646_). Laser power was optimized for single molecule movie recording so that >90% molecules were bleached at the end of each movie. For the measurement of Halo-tagged fluorophores, laser powers of approximately 6.6 mW mm^−2^ for 561 nm and 6.8 mW mm^−2^ for 640 nm were used. For the measurement of SNAP-tagged fluorophores, laser powers of approximately 1.5 mW mm^−2^ for 561 nm and 1.9 W mm^−2^ for 640 nm were used. Power output was measured at the objective using a Thor Lab PM100D Optic Power Meter.

To analyze the intensity and stability of dyes, we first used SPARTAN^44^ software to manually examine single molecule fluorescence traces and isolated traces showing only single step photobleaching. Single molecule intensity was then measured by averaging the first 5 frames from each trace per movie to construct histograms. For measurement of fluorophore stability, we measured the time before each fluorescence single molecule trace photobleaching across movies. We then calculated the τ by fitting the survival plot with a single exponential function. OriginPro software was used to plot histograms and survival plots and for fitting.

### Cell culture, staining and microscopy

#### HEK293T

HEK293T cells were cultured in growth medium (DMEM, Glutamax, 4.5 g Glucose, 10% FCS, 1% PS; Invitrogen) at 37 °C and 5% CO_2_. 30 000 cells per well were seeded on 8-well μL slides (Ibidi) previously coated with 0.25 mg ml^−1^ poly-l-lysine (Aldrich, mol wt 70 000–150 000). The next day, 400 ng DNA was transfected using 0.8 μL Jet Prime reagent in 40 μL Jet Prime buffer (VWR) per well. Medium was exchanged against antibiotic-free media before the transfection mix was pipetted on the cells. After 4 hours incubation at 37 °C and 5% CO_2_, medium was exchanged against growth media. After 24 hours cells were stained and imaged. All dyes were used at a concentration of 100 nM. 5 μM Hoechst 33342 was used to stain DNA. Staining was done in growth medium at 37 °C, 5% CO_2_ for 30 minutes. Afterwards cells were washed once in growth media and imaged live in cell imaging buffer (Invitrogen) using an epifluorescence microscope, Nikon Ti-E equipped with pE4000 (cool LED), Penta Cube (AHF 66-615), 60× oil NA 1.49 (Apo TIRF Nikon) and imaged on a sCMOS camera (Prime 95B, Photometrics) operated by NIS Elements (Nikon). For excitation the following LED wavelengths were used: Hoechst – 405 nm, JF_549_ and Sulfo549 – 550 nm, JF_646_ and Sulfo646 – 635 nm.

HA-Halo or HA-SNAP tagged β_2_AR were expressed in HEK293T cells in media containing 5% FBS at 37 °C, 5% CO_2_ on 18 mm poly-l-lysine-coated coverslips in a 12-well plate. Total 0.7 μg of β_2_AR plasmids were transfected using Lipofectamine 2000 (Thermo Scientific). 6–8 hours after transfection, cellular media was exchanged with fresh media. ∼24 hours post-transfection, cells were washed with extracellular buffer containing (in mM): 10 HEPES, 135 NaCl, 5.4 KCl, 2 CaCl_2_, 1 MgCl_2_, pH 7.4 and immersed in a labelling solution containing 2 μM dye for 45 minutes at 37 °C. Unbound excess fluorophores were washed with EX solution for >30 minutes, labelled cells on a coverslip were imaged using a 60× objective (NA. 1.49) on an inverted microscope (Olympus IX83) for live cell imaging. 561 nm or 640 nm lasers was used to excite Sulfo549 (or JF_549_) or Sulfo646 (or JF_646_), respectively.

#### AD293

AD293 cells were cultured in DMEM (D6546, Merck) supplemented with 10% FCS (Merck) and 2 mM l-glutamine (Thermo Scientific) at 37 °C and 5% CO_2_. Cells were seeded on poly-l-lysine-coated (MW > 300 000, 0.1 mg ml^−1^, Biochrom) 8-well chamber slides (Nunc Lab-Tek II) and transfected the next day (50–70% confluency). For transfection, growth media was exchanged against Opti-MEM (Gibco), and 110 ng plasmid DNA (SNAP-GLP1R from Cisbio, for sequence of Halo-GLP1R see ESI†) and 0.3 μL Lipofectamine 2000 (Invitrogen) per chamber were mixed in Opti-MEM, incubated for 5 minutes and then added to the cells. After incubation at 37 °C and 5% CO_2_ for 5 hours, Opti-MEM was exchanged against growth media. AD293 cells were labelled and imaged 24 hours after transfection. Cells were labelled in growth media containing 500 nM of dyes for 30 minutes at 37 °C and 5% CO_2_. For the last 5 minutes of incubation, Hoechst33342 (4.4 μM) was added. After one wash, cells were imaged in growth media using a Nikon Ti-E automated base and 60×/1.4 NA objective. Excitation was delivered at *λ* = 395/25 nm (Hoechst), 575/25 nm (JF_549_ and Sulfo549), and 640/30 nm (JF_646_ and Sulfo646) using a Lumencor Spectra X Light engine, and emitted signals were detected at *λ* = 460/50 nm, 630/75 nm, and 700/75 nm, respectively, using a Photometrics Evolve Delta 512 EMCCD.

#### Human iPSC-derived neurons

Human induced pluripotent stem cells (iPSCs) engineered to express mNGN2 under a doxycycline-inducible system in the AAVS1 safe harbor locus were used for the i^3^Neuron differentiation protocol, as described previously.^23,24^ In brief, iPSCs were seeded on Matrigel (Corning)-coated dishes in StemFlex Medium (Gibco) supplemented with 5 nM Y-27632 dihydrochloride ROCK inhibitor (Stem Cell Technologies). The iPSCs were subsequently fed daily for 3 consecutive days with Neuronal Induction Medium (DMEM/F12 medium (Gibco) containing 2.5 μg mL^−1^ doxycycline, 1× N2-supplement (Gibco), 1× NEAA (Gibco), 1× GlutaMAX (Gibco), 1× Pen/Strep (Gibco), 10 ng mL^−1^ BDNF (PeproTech), 10 ng mL^−1^ NT-3 (PeproTech) and 1 μg mL^−1^ L aminin (Gibco)). On the third day, Neuronal Induction Medium was supplemented with 6 μg ml^−1^ puromycin. After 3 days, pre-differentiated i^3^Neurons were dispersed using Trypsin (Gibco) and co-cultured with primary murine astrocytes on matrigel-coated 25 mm glass coverslips in Neuron Culture Medium (NeuroBasal Medium (Gibco), supplemented with 1× B27 (Gibco), 1× GlutaMAX (Gibco), 1× Pen/Strep (Gibco), 10 ng mL^−1^ BDNF (PeproTech), 10 ng mL^−1^ NT-3 (PeproTech) and 1 μg mL^−1^ Laminin (Gibco)). 50% of the Neuron Culture Medium was replaced every 2–3 days and supplemented with 2 μM araC 5 days after culturing, to limit glial proliferation. i^3^Neurons were transfected, labelled and imaged as described below 10–12 days after co-culturing.

#### Neuronal transfection, labelling and imaging

i^3^Neurons were transfected with 2 μg of plasmid (SNAP-TM-Halo or Halo-TM-SNAP) per 25 mm coverslip using a calcium phosphate transfection kit (Promega), according to manufacturer's instruction. 24 hours after transfection, cells were labelled with 500 nM in parallel of either BG-Sulfo646 and Halo-JF_549_, or Halo-Sulfo646 and BG-JF_549_, dissolved in Neuron Culture Medium for 30 minutes at 37 °C.

For live imaging, i^3^Neurons were washed once with Neuron Culture Medium before imaging in conditioned Neuron Culture Medium using a spinning disc confocal microscope (Ti Eclipse, Nikon) equipped with a spinning disk (CSU-X1, Yokogawa), EMCCD Camera (AU-888, Andor), 60× Plan-Apo NA 1.40 objective (oil immersion, Nikon), incubation chamber (37 °C, 5% CO_2_, Okolab). JF_549_ and Sulfo646 were excited with 561 nm and 638 nm lasers, respectively, and emission was detected within 600–650 and 700–775 nm filter range, respectively. Images were taken using an additional 1× lens, resulting in 110 nm effective pixel size.

For super-resolution imaging, labelled i^3^Neurons were washed once with PBS before fixing 20 minutes at room temperature with 4% PFA and 4% sucrose in PBS. Fixation solution was removed and fixed i^3^Neurons were incubated with quenching solution (0.1 M glycine, 0.1 M NH_4_Cl in PBS) for 10 minutes at room temperature. Coverslips were subsequently washed once with PBS and once with water, mounted in ProLong Gold Antifade (ThermoFisher), and cured for 24 hours at room temperature. STED imaging was performed on a Leica TCS 3× gSTED microscope, equipped with a pulsed white light excitation laser (NKT Photonics) and a 775 nm depletion laser. Two-channel STED imaging was performed by sequentially exciting JF_549_ or Sulfo646 at 550 and 640 nm, respectively, using a 100× PL Apo NA1.4 objective (oil immersion, Leica). The 775 nm STED laser was used to deplete both JF_549_ and Sulfo646. Time-gated detection was set from 0.5–6 ns for all dyes and emission was detected within 560–643 and 650–751 nm, respectively. Fluorescence signal was detected sequentially by two hybrid detectors, 6-fold zoom, 8-bit sampling and 1024 × 1024 pixel scanning format, resulting in 18.9 × 18.9 nm pixel dimension.

## Ethical statement

The human iPSC-derived cell line (BIHi005-A) was generated following authorization by the donor and ethics approval from the primary project and has been registered in the European Human Pluripotent Stem Cell Registry (hPSCreg): http://hpscreg.eu/cell-line/BIHi005-A. Cells were kindly provided by Dr Sebastian Diecke, MDC, Berlin, Germany. Preparation of primary murine astrocytes (C57BL/6) was reviewed and approved by the ethics committee of the “Landesamt für Gesundheit und Soziales” (LAGeSo) Berlin and were conducted accordingly to the committee's guidelines.

## Author contributions

JB designed and conceptualized the study. BM, KR, CH, and JB performed chemical synthesis and characterization. JLee performed SiMPull. JA, DR, JLee, MLehmann, and JB performed microscopy. MLisurek, DB and HS performed modelling and docking. BJ provided reagents. MLehmann, JLevitz, VH, DJH, and JB supervised the study. JLevitz, DJH and JB wrote the manuscript with input from all the authors.

## Conflicts of interest

None declared.

## Supplementary Material

OB-020-D1OB02216D-s001
