# Supplementary material for: Sulfonated red and far-red rhodamines to visualize SNAP- and Halo-tagged cell surface proteins
Source: Org Biomol Chem. 2022 Feb 21;20(30):5967–80. doi: 10.1039/d1ob02216d (PMC9346974; doi:10.1039/d1ob02216d)
Supplement: OB-020-D1OB02216D-s001 [file OB-020-D1OB02216D-s001.pdf]

Electronic Supplementary Material (ESI) for Organic & Biomolecular Chemistry.
This journal is © The Royal Society of Chemistry 2022

Supporting Information

Sulfonated red and far-red rhodamines to visualize SNAP- and Halo-tagged cell surface proteins

Ramona Birke[1], Julia Ast[2,3,4], Dorien A. Roosen[5], Joon Lee[6], Kilian Roßmann[1], Christiane Huhn[1], Bettina Mathes[7], Michael Lisurek[8], David Bushiri[8], Han Sun[8], Ben Jones[9], Martin Lehmann[5], Joshua Levitz[6], Volker Haucke[5], David J. Hodson[2,3,4,10,*], and Johannes Broichhagen[1,7,*]

[1] Leibniz-Forschungsinstitut für Molekulare Pharmakologie, Berlin, Germany.

[2] Institute of Metabolism and Systems Research (IMSR), University of Birmingham, Birmingham, B15 2TT, UK.

[3] Centre for Endocrinology, Diabetes and Metabolism, Birmingham Health Partners, Birmingham, B15 2TT, UK.

[4] Centre of Membrane Proteins and Receptors (COMPARE), University of Birmingham, Birmingham, UK.

[5] Department of Molecular Pharmacology and Cell Biology, Leibniz-Forschungsinstitut für Molekulare Pharmakologie, Berlin, Germany.

[6] Department of Biochemistry, Weill Cornell Medicine, New York, NY 10065

[7] Department of Chemical Biology, Max Planck Institute for Medical Research, Heidelberg, Germany.

[8] Structural Chemistry and Computational Biophysics, Leibniz-Forschungsinstitut für Molekulare Pharmakologie, Berlin, Germany.

[9] Section of Endocrinology and Investigative Medicine, Imperial College London, London W12 0NN, United Kingdom.

[10] Oxford Centre for Diabetes, Endocrinology and Metabolism, Radcliffe Department of Medicine, University of Oxford, Churchill Hospital, Oxford, UK

* correspondence should be addressed to: d.hodson@bham.ac.uk, broichhagen@fmp-berlin.de

Table of Contents

1. General 4

2. Synthesis 5

2.1. General procedure A for Buchwald-Hartwig coupling and deprotection 5

2.2. General procedure B for taurine coupling and deprotection 5

2.3. General procedure C for peptide coupling with TSTU 5

2.4. 4-(tert-Butoxycarbonyl)-2-(3-(3-carboxyazetidin-1-ium-1-ylidene)-6-(3-carboxy-azetidin-1-yl)-3H-xanthen-9-yl)benzoate (2a) 6

2.5. 4-Carboxy-2-(3-(3-((2-sulfoethyl)carbamoyl)azetidin-1-ium-1-ylidene)-6-(3-((2-sulfoethyl)carbamoyl)azetidin-1-yl)-3H-xanthen-9-yl)benzoate (3a) 6

2.6. 4-((4-(((2-Amino-9H-purin-6-yl)oxy)methyl)benzyl)carbamoyl)-2-(3-(3-((2-sulfoethyl)carbamoyl)azetidin-1-ium-1-ylidene)-6-(3-((2-sulfoethyl)carbamoyl)-azetidin-1-yl)-3H-xanthen-9-yl)benzoate (BG-Sulfo549) 7

2.7. 4-((2-(2-((6-Chlorohexyl)oxy)ethoxy)ethyl)carbamoyl)-2-(3-(3-((2-sulfoethyl)-carbamoyl)azetidin-1-ium-1-ylidene)-6-(3-((2-sulfoethyl)carbamoyl)azetidin-1-yl)-3H-xanthen-9-yl)benzoate (CA-Sulfo549) 7

2.8. 4-(tert-Butoxycarbonyl)-2-(3-(3-carboxyazetidin-1-ium-1-ylidene)-7-(3-carboxy-azetidin-1-yl)-5,5-dimethyl-3,5-dihydrodibenzo[b,e]silin-10-yl)benzoate (2b) 8

2.9. 4-Carboxy-2-(5,5-dimethyl-3-(3-((2-sulfoethyl)carbamoyl)azetidin-1-ium-1-ylidene)-7-(3-((2-sulfoethyl)carbamoyl)azetidin-1-yl)-3,5-dihydrodibenzo[b,e]silin-10-yl)benzoate (3b) 8

2.10. 4-((4-(2-(2-Amino-9H-purin-6-yl)ethyl)benzyl)carbamoyl)-2-(5,5-dimethyl-3-(3-((2-sulfoethyl)carbamoyl)azetidin-1-ium-1-ylidene)-7-(3-((2-sulfoethyl)-carbamoyl)azetidin-1-yl)-3,5-dihydrodibenzo[b,e]silin-10-yl)benzoate (BG-Sulfo646) 9

2.11. 4-((2-(2-((6-Chlorohexyl)oxy)ethoxy)ethyl)carbamoyl)-2-(5,5-dimethyl-3-(3-((2-sulfoethyl)carbamoyl)azetidin-1-ium-1-ylidene)-7-(3-((2-sulfoethyl)-carbamoyl)azetidin-1-yl)-3,5-dihydrodibenzo[b,e]silin-10-yl)benzoate (CA-Sulfo646) 9

3. NMR spectra 10

3.1. 4-(tert-Butoxycarbonyl)-2-(3-(3-carboxyazetidin-1-ium-1-ylidene)-6-(3-carboxy-azetidin-1-yl)-3H-xanthen-9-yl)benzoate (2a) 10

3.2. 4-Carboxy-2-(3-(3-((2-sulfoethyl)carbamoyl)azetidin-1-ium-1-ylidene)-6-(3-((2-sulfoethyl)carbamoyl)azetidin-1-yl)-3H-xanthen-9-yl)benzoate (3a) 11

3.3. 4-Carboxy-2-(3-(3-((2-sulfoethyl)carbamoyl)azetidin-1-ium-1-ylidene)-6-(3-((2-sulfoethyl)carbamoyl)azetidin-1-yl)-3H-xanthen-9-yl)benzoate (3a)4-((4-(((2-Amino-9H-purin-6-yl)oxy)methyl)benzyl)carbamoyl)-2-(3-(3-((2-sulfoethyl)carbamoyl)azetidin-1-ium-1-ylidene)-6-(3-((2-sulfoethyl)carbamoyl)-azetidin-1-yl)-3H-xanthen-9-yl)benzoate (BG-Sulfo549) 12

3.4. 4-((2-(2-((6-Chlorohexyl)oxy)ethoxy)ethyl)carbamoyl)-2-(3-(3-((2-sulfoethyl)-carbamoyl)azetidin-1-ium-1-ylidene)-6-(3-((2-sulfoethyl)carbamoyl)azetidin-1-yl)-3H-xanthen-9-yl)benzoate (CA-Sulfo549) 13

3.5. 4-(tert-Butoxycarbonyl)-2-(3-(3-carboxyazetidin-1-ium-1-ylidene)-7-(3-carboxy-azetidin-1-yl)-5,5-dimethyl-3,5-dihydrodibenzo[b,e]silin-10-yl)benzoate (2b) 14

3.6. 4-Carboxy-2-(5,5-dimethyl-3-(3-((2-sulfoethyl)carbamoyl)azetidin-1-ium-1-ylidene)-7-(3-((2-sulfoethyl)carbamoyl)azetidin-1-yl)-3,5-dihydrodibenzo[b,e]silin-10-yl)benzoate (3b) 15

3.7. 4-((4-(2-(2-Amino-9H-purin-6-yl)ethyl)benzyl)carbamoyl)-2-(5,5-dimethyl-3-(3-((2-sulfoethyl)carbamoyl)azetidin-1-ium-1-ylidene)-7-(3-((2-sulfoethyl)-carbamoyl)azetidin-1-yl)-3,5-dihydrodibenzo[b,e]silin-10-yl)benzoate (BG-Sulfo646) 16

3.8. 4-((2-(2-((6-Chlorohexyl)oxy)ethoxy)ethyl)carbamoyl)-2-(5,5-dimethyl-3-(3-((2-sulfoethyl)carbamoyl)azetidin-1-ium-1-ylidene)-7-(3-((2-sulfoethyl)-carbamoyl)azetidin-1-yl)-3,5-dihydrodibenzo[b,e]silin-10-yl)benzoate (CA-Sulfo646) *17*

4. Protein mass spectrometry 18

5. Protein constructs 19

6. Supplemental Figures 20

7. Supplemental Schemes 25

8. References 26

1. General

All chemical reagents and anhydrous solvents for synthesis were purchased from commercial suppliers (AAT Bioquest, Roth, Sigma-Aldrich, Fluka, Acros, Fluorochem, TCI) and were used without further purification or distillation.

BG-NH$_2$ and **CA-NH$_2$** were prepared and obtained as described previously.[1]

NMR spectra were recorded in deuterated solvents on a Bruker AVANCE III 600 and calibrated to residual solvent peaks (^{1}H in ppm): MeOD-d$_4$ (3.31). Multiplicities are abbreviated as follows: s = singlet, d = doublet, t = triplet, q = quartet, p = pentet, br = broad, m = multiplet. Coupling constants *J* are reported in Hz. Spectra are reported based on appearance, not on theoretical multiplicities derived from structural information.

LC-MS was performed on an Agilent 1260 Infinity II LC System equipped with Agilent SB-C18 column (1.8 µm, 2.1 × 50 mm). Buffer A: 0.1% FA in H_2O Buffer B: 0.1% FA acetonitrile. The typical gradient was from 10% B for 0.5 min → gradient to 95% B over 5 min → 95% B for 0.5 min → gradient to 99% B over 1 min with 0.8 mL/min flow.

High resolution mass spectrometry was performed using a Bruker maXis II ETD hyphenated with a Shimadzu Nexera system. The instruments were controlled via Brukers otofControl 4.1 and Hystar 4.1 SR2 (4.1.31.1) software. The acquisition rate was set to 3 Hz and the following source parameters were used for positive mode electrospray ionization: End plate offset = 500 V; capillary voltage = 3800 V; nebulizer gas pressure = 45 psi; dry gas flow = 10 L/min; dry temperature = 250 °C. Transfer, quadrupole and collision cell settings are mass range dependent and were fine-adjusted with consideration of the respective analyte's molecular weight. For internal calibration sodium format clusters were used. Samples were desalted via fast liquid chromatography. A Supelco Titan™ C18 UHPLC Column, 1.9 µm, 80 Å pore size, 20 × 2.1 mm and a 2 min gradient from 10 to 98% aqueous MeCN with 0.1% FA (H_2O: Carl Roth GmbH + Co. KG ROTISOLV® Ultra LC-MS; MeCN: Merck KGaA LiChrosolv® Acetonitrile hypergrade for LC-MS; FA - Merck KGaA LiChropur® Formic acid 98%-100% for LC-MS) was used for separation. Sample dilution in 10% aqueous ACN (hyper grade) and injection volumes were chosen dependent of the analyte's ionization efficiency. Hence, on-column loadings resulted between 0.25–5.0 ng. Automated internal re-calibration and data analysis of the recorded spectra were performed with Bruker's DataAnalysis 4.4 SR1 software.

Preparative or semi-preparative HPLC was performed an Agilent 1260 Infinity II LC System equipped with columns as followed: preparative column –Reprospher 100 C18 columns (10 µm: 50 x 30 mm at 20 mL/min flow rate; semi-preparative column – 5 µm: 250 x 10 mm at 4 mL/min flow rate. Eluents A (0.1% TFA in H_2O) and B (0.1% TFA in MeCN) were applied as a linear gradient. Peak detection was performed at maximal absorbance wavelength.

Abbreviations: Boc: *tert*-butoxycarbonyl; DIPEA: *N,N*-diisopropylethylamine; DMSO: dimethylsulfoxide; FA: formic acid; HBTU: (2-(1*H*-benzotriazol-1-yl)-1,1,3,3-tetramethyluronium hexafluorophosphate; TFA: trifluoroacetic acid; TSTU: *N,N,N',N'*-Tetramethyl-*O*-(*N*-succinimidyl)uronium tetrafluoroborate.

2. Synthesis

2.1. General procedure A for Buchwald-Hartwig coupling and deprotection

A flame-dried Schlenk flask was charged with bistriflate **1** (AAT Bioquest, 1.0 equiv.), $Pd_2(dba)_3$ (0.2 equiv.), XPhos (0.3 equiv.), Cs_2CO_3 (4.8 equiv.), and dissolved in dry 1,4-dioxanes (345 equiv.) under an argon atmosphere. Methyl azetidine 3-carboxylate hydrochloride (3.0 equiv.) was added and the reaction mixture was heated to 80 °C for 4 h. After cooling, MeOH (345 equiv.) and 1 M LiOH (345 equiv.) were added and the reaction was allowed to stir for 1 h at r.t. before it was quenched with 1 mL HOAc. All volatiles were removed in vacuo, the residue taken up in DMSO:MeCN:H_2O:HOAc (2:1:1:0.02), filtered using a 0.45 μm syringe filter and subjected to RP-HPLC purification.

2.2. General procedure B for taurine coupling and deprotection

A 4 mL dram vial was charged with bisacid **2** (1.0 equiv.) dissolved in DMF (2 mg/1 mL) and DIPEA (8.0 equiv.) and TSTU (2.8 equiv.) were combined before taurine (3.0 equiv.) was added in one portion. The reaction mixture was allowed to incubate for 1 h before it was quenched by addition of HOAc (10.0 equiv.) and 10 vol% water and subjected to RP-HPLC purification. The product containing fractions were pooled and lyophilized, and upon dryness redissolved in neat TFA and incubated for 3 h at r.t. before all volatiles were removed under a gentle stream of nitrogen, before subjecting to RP-HPLC purification. The product containing fractions were pooled and lyophilized.

2.3. General procedure C for peptide coupling with TSTU

A 4 mL dram vial was charged with acid **3** (1.0 equiv.) dissolved in DMF (2 mg/1 mL) and DIPEA (8.0 equiv.) and TSTU (1.4 equiv.) were combined before either **BG-NH$_2$** or **CA-NH$_2$** (1.5 equiv.) was added in one portion. The reaction mixture was allowed to incubate for 1 h before it was quenched by addition of HOAc (10.0 equiv.) and 10 vol% water and subjected to RP-HPLC purification. The product containing fractions were pooled and lyophilized to obtain the desired product.

2.4. 4-(*tert*-Butoxycarbonyl)-2-(3-(3-carboxyazetidin-1-ium-1-ylidene)-6-(3-carboxy-azetidin-1-yl)-3*H*-xanthen-9-yl)benzoate (2a)

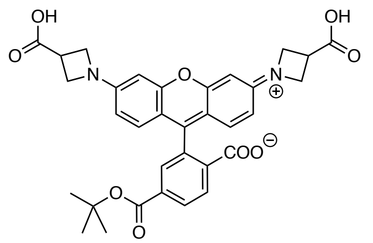

2a was prepared according to general procedure A.
bistriflate: **1a** (150 mg, 215 µmol), yield: 95 mg (74%). Red powder.
^{1}H NMR (600 MHz, MeOD-d$_4$): δ [ppm] = 8.40 (d, J = 8.2 Hz, 1H), 8.33 (dd, J = 8.2, 1.7 Hz, 1H), 7.89 (d, J = 1.7 Hz, 1H), 7.11 (m, 2H), 6.77-6.56 (m, 4H), 4.50 (dd, J = 10.4, 8.6 Hz, 4H), 4.42 (dd, J = 10.6, 5.9 Hz, 4H), 3.78-3.54 (m, 2H), 1.59 (s, 9H).
HRMS (ESI): calc. for $C_{33}H_{31}N_2O_9$ $[M+H]^+$: 599.2024, found: 599.2021.

2.5. 4-Carboxy-2-(3-(3-((2-sulfoethyl)carbamoyl)azetidin-1-ium-1-ylidene)-6-(3-((2-sulfoethyl)carbamoyl)azetidin-1-yl)-3*H*-xanthen-9-yl)benzoate (3a)

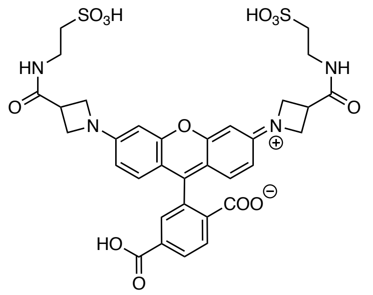

3a was prepared according to general procedure B.
acid: **2a** (20 mg, 33.4 µmol), yield: 12.5 mg (49%). Red powder.
^{1}H NMR (600 MHz, MeOD-d$_4$): δ [ppm] = 8.60-8.19 (m, 2H), 7.98 (d, J = 1.7 Hz, 1H), 7.10 (d, J = 9.1 Hz, 2H), 6.80-6.34 (m, 4H), 4.42 (m, 4H), 4.37 (m, 4H), 3.67-3.60 (m, 6H), 3.00 (t, J = 6.6 Hz, 4H).
HRMS (ESI): calc. for $C_{33}H_{33}N_4O_{13}S_2$ $[M+H]^+$: 757.1480, found: 757.1476.

2.6. 4-((4-(((2-Amino-9*H*-purin-6-yl)oxy)methyl)benzyl)carbamoyl)-2-(3-(3-((2-sulfoethyl)carbamoyl)azetidin-1-ium-1-ylidene)-6-(3-((2-sulfoethyl)carbamoyl)-azetidin-1-yl)-3*H*-xanthen-9-yl)benzoate (BG-Sulfo549)

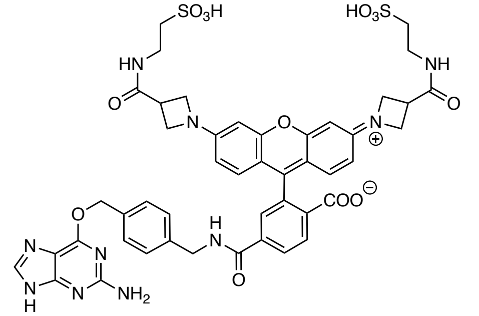

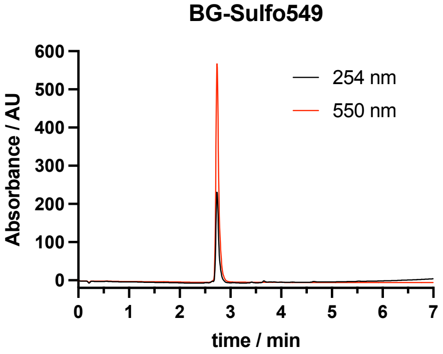

BG-Sulfo549 was prepared according to general procedure C.
amine: **BG-NH$_2$**
acid: **3a** (6.0 mg, 7.9 μmol), yield: 3.0 mg (38%). Red powder.
^{1}H NMR (600 MHz, DMSO-d$_6$): δ [ppm] = 9.29 (s, 1H), 8.65 (s, 1H), 8.30 (d, *J* = 8.3 Hz, 1H), 8.25 (d, *J* = 9.3 Hz, 1H), 8.09 (s, 2H), 7.89 (s, 1H), 7.50 (d, *J* = 8.0 Hz, 2H), 7.36 (d, *J* = 8.0 Hz, 2H), 7.00 (d, *J* = 9.0 Hz, 2H), 6.65 (d, *J* = 9.2 Hz, 2H), 6.63 (s, 1H), 5.55 (s, 2H), 4.50-4.46 (m, 2H), 4.40-4.32 (m, 4H), 4.28-4.20 (m, 4H), 2.60 (t, *J* = 7.2 Hz, 4H). (some protons masked under water peak).
HRMS (ESI): calc. for $C_{46}H_{46}N_{10}O_{13}S_2$ $[M+2H]^{2+}$: 505.1338, found: 505.1336.

2.7. 4-((2-(2-((6-Chlorohexyl)oxy)ethoxy)ethyl)carbamoyl)-2-(3-(3-((2-sulfoethyl)-carbamoyl)azetidin-1-ium-1-ylidene)-6-(3-((2-sulfoethyl)carbamoyl)azetidin-1-yl)-3*H*-xanthen-9-yl)benzoate (CA-Sulfo549)

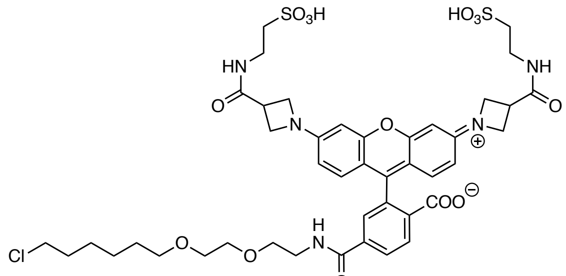

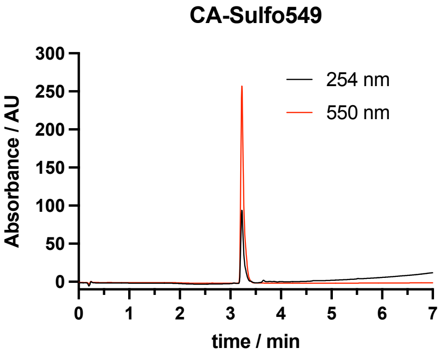

CA-Sulfo549 was prepared according to general procedure C.
amine: **Halo-NH$_2$**
acid: **3a** (6.0 mg, 7.9 μmol), yield: 4.5 mg (59%). Red powder.
^{1}H NMR (600 MHz, MeOD-d$_4$): δ [ppm] = 8.42 (d, *J* = 8.2 Hz, 1H), 8.23 (dd, *J* = 8.3, 1.8 Hz, 1H), 7.85 (s, 1H), 7.12 (s, 1H), 7.10 (s, 1H), 6.67 (dd, *J* = 9.2, 2.2 Hz, 2H), 6.57 (d, *J* = 2.1 Hz, 2H), 4.42 (q, *J* = 8.5 Hz, 4H), 4.37 (q, *J* = 4.8 Hz, 4H), 3.69-3.62 (m, 10H), 3.62-3.56 (m, 4H), 3.55 (t, *J* = 6.6 Hz, 2H), 3.45 (t, *J* = 6.5 Hz, 2H), 3.03 (t, *J* = 6.6 Hz, 4H), 1.74 (p, *J* = 7.1 Hz, 2H), 1.53 (p, *J* = 7.0 Hz, 2H), 1.42 (p, *J* = 5.0 Hz, 2H), 1.38-1.30 (m, 2H).
HRMS (ESI): calc. for $C_{43}H_{53}ClN_5O_{14}S_2$ $[M+H]^+$: 962.2713, found: 962.2721.

2.8. 4-(*tert*-Butoxycarbonyl)-2-(3-(3-carboxyazetidin-1-ium-1-ylidene)-7-(3-carboxy-azetidin-1-yl)-5,5-dimethyl-3,5-dihydrodibenzo[*b*,*e*]silin-10-yl)benzoate (2b)

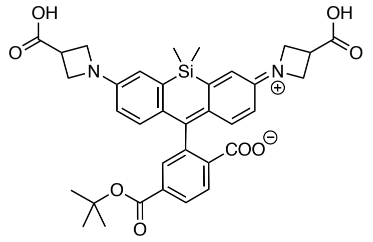

2b was prepared according to general procedure A.
bistriflate: **1b** (139 mg, 203 μmol), yield: 20.0 mg (37%). Blue powder.
^{1}H NMR (600 MHz, MeOD-d$_4$): *δ* [ppm] = 8.35-8.01 (m, 2H), 7.71 (d, *J* = 1.1 Hz, 1H), 7.01–6.71 (m, 4H), 6.40 (dd, *J* = 9.1, 2.6 Hz, 2H), 4.44-4.09 (m, 8H), 3.74-3.48 (m, 2H), 1.57 (s, 9H), 0.64 (s, 3H), 0.55 (s, 3H).
HRMS (ESI): calc. for $C_{35}H_{37}N_2O_8Si$ $[M+H]^+$: 641.2314, found: 641.2314.

2.9. 4-Carboxy-2-(5,5-dimethyl-3-(3-((2-sulfoethyl)carbamoyl)azetidin-1-ium-1-ylidene)-7-(3-((2-sulfoethyl)carbamoyl)azetidin-1-yl)-3,5-dihydrodibenzo[*b*,*e*]silin-10-yl)benzoate (3b)

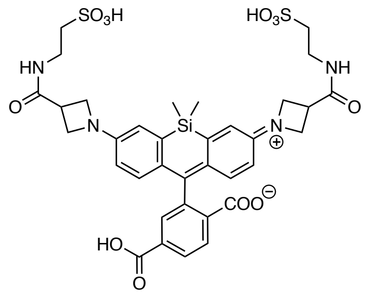

3b was prepared according to general procedure B.
acid: **2b** (20.0 mg, 31.3 μmol), yield: 12.5 mg (50%). Blue powder.
^{1}H NMR (600 MHz, MeOD-d$_4$): *δ* [ppm] = 8.36-8.14 (m, 2H), 7.81 (d, *J* = 1.7 Hz, 1H), 7.05-6.70 (m, 4H), 6.43-6.19 (m, 2H), 4.59-4.22 (m, 8H), 3.64 (t, *J* = 6.6 Hz, 4H), 3.00 (t, *J* = 6.6 Hz, 4H), 0.61 (s, 3H), 0.54 (s, 3H).
HRMS (ESI): calc. for $C_{35}H_{39}N_4O_{12}S_2Si$ $[M+H]^+$: 799.1770, found: 799.1767.

2.10. 4-((4-(2-(2-Amino-9*H*-purin-6-yl)ethyl)benzyl)carbamoyl)-2-(5,5-dimethyl-3-(3-((2-sulfoethyl)carbamoyl)azetidin-1-ium-1-ylidene)-7-(3-((2-sulfoethyl)-carbamoyl)azetidin-1-yl)-3,5-dihydrodibenzo[*b*,*e*]silin-10-yl)benzoate (BG-Sulfo646)

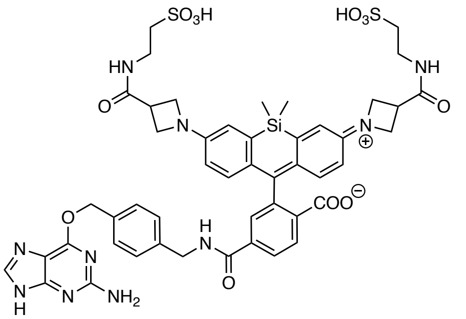

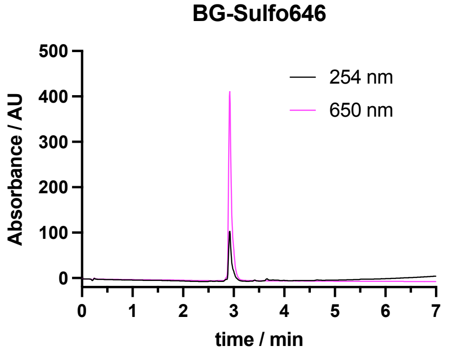

BG-Sulfo646 was prepared according to general procedure C.
amine: **BG-NH$_2$**
acid: **3b** (6.0 mg, 7.5 μmol), yield: 2.5 mg (32%). Blue powder.
^{1}H NMR (600 MHz, DMSO-d$_6$): δ [ppm] = 9.30 (t, J = 5.8 Hz 1H), 8.12 (d, J = 7.9 Hz, 1H), 8.04 (d, J = 7.8 Hz, 1H), 7.94 (s, 2H), 7.70 (s, 1H), 7.50 (d, J = 7.9 Hz, 2H), 7.34 (d, J = 7.9 Hz, 2H), 6.75 (s, 2H), 6.63 (d, J = 8.7 Hz, 2H), 6.63 (d, J = 8.7 Hz, 2H), 6.36 (d, J = 8.7 Hz, 2H), 5.58 (s, 2H), 4.44 (d, J = 5.7 Hz, 2H), 4.06-3.93 (m, 4H), 3.88-3.77 (m, 4H), 2.61-2.56 (m, 6H), 2.09 (s, 1H), 2.08 (s, 1H), 1.92 (s, 1H), 1.24 (s, 4H), 0.60 (s, 3H), 0.49 (s, 3H).
HRMS (ESI): calc. for $C_{48}H_{52}N_{10}O_{12}S_2Si$ $[M+2H]^{2+}$: 526.1483, found: 526.1481.

2.11. 4-((2-(2-((6-Chlorohexyl)oxy)ethoxy)ethyl)carbamoyl)-2-(5,5-dimethyl-3-(3-((2-sulfoethyl)carbamoyl)azetidin-1-ium-1-ylidene)-7-(3-((2-sulfoethyl)-carbamoyl)azetidin-1-yl)-3,5-dihydrodibenzo[*b*,*e*]silin-10-yl)benzoate (CA-Sulfo646)

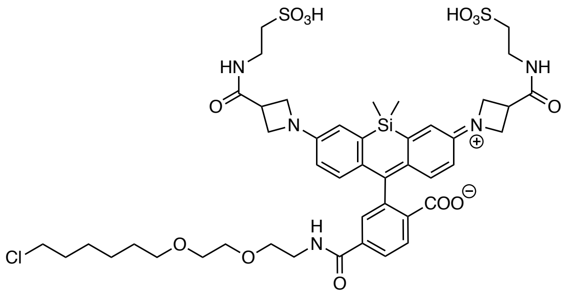

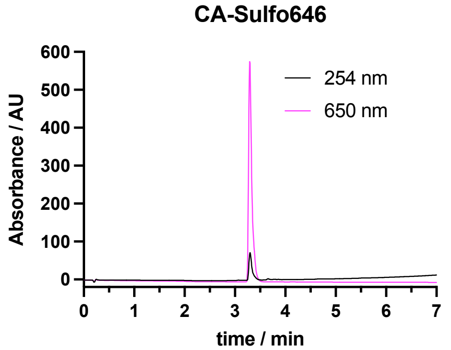

CA-Sulfo646 was prepared according to general procedure C.
amine: **Halo-NH$_2$**
acid: **3b** (6.0 mg, 7.5 μmol), yield: 5.2 mg (69%). Blue powder.
^{1}H NMR (600 MHz, MeOD-d$_4$): δ [ppm] = 8.42 (d, J = 8.2 Hz, 1H), 8.23 (dd, J = 8.3, 1.8 Hz, 1H), 7.85 (s, 1H), 7.12 (s, 1H), 7.10 (s, 1H), 6.67 (dd, J = 9.2, 2.2 Hz, 2H), 6.57 (d, J = 2.1 Hz, 2H), 4.42 (q, J = 8.5 Hz, 4H), 4.37 (q, J = 4.8 Hz, 4H), 3.69-3.62 (m, 10H), 3.62-3.56 (m, 4H), 3.55 (t, J = 6.6 Hz, 2H), 3.45 (t, J = 6.5 Hz, 2H), 3.03 (t, J = 6.6 Hz, 4H), 1.74 (p, J = 7.1 Hz, 2H), 1.53 (p, J = 7.0 Hz, 2H), 1.42 (p, J = 5.0 Hz, 2H), 1.38-1.30 (m, 2H).
HRMS (ESI): calc. for $C_{45}H_{56}ClN_5O_{13}S_2Si$ $[M-2H]^{2-}$: 500.6392, found: 500.6390.

3. NMR spectra

3.1. 4-(*tert*-Butoxycarbonyl)-2-(3-(3-carboxyazetidin-1-ium-1-ylidene)-6-(3-carboxy-azetidin-1-yl)-3*H*-xanthen-9-yl)benzoate (2a)

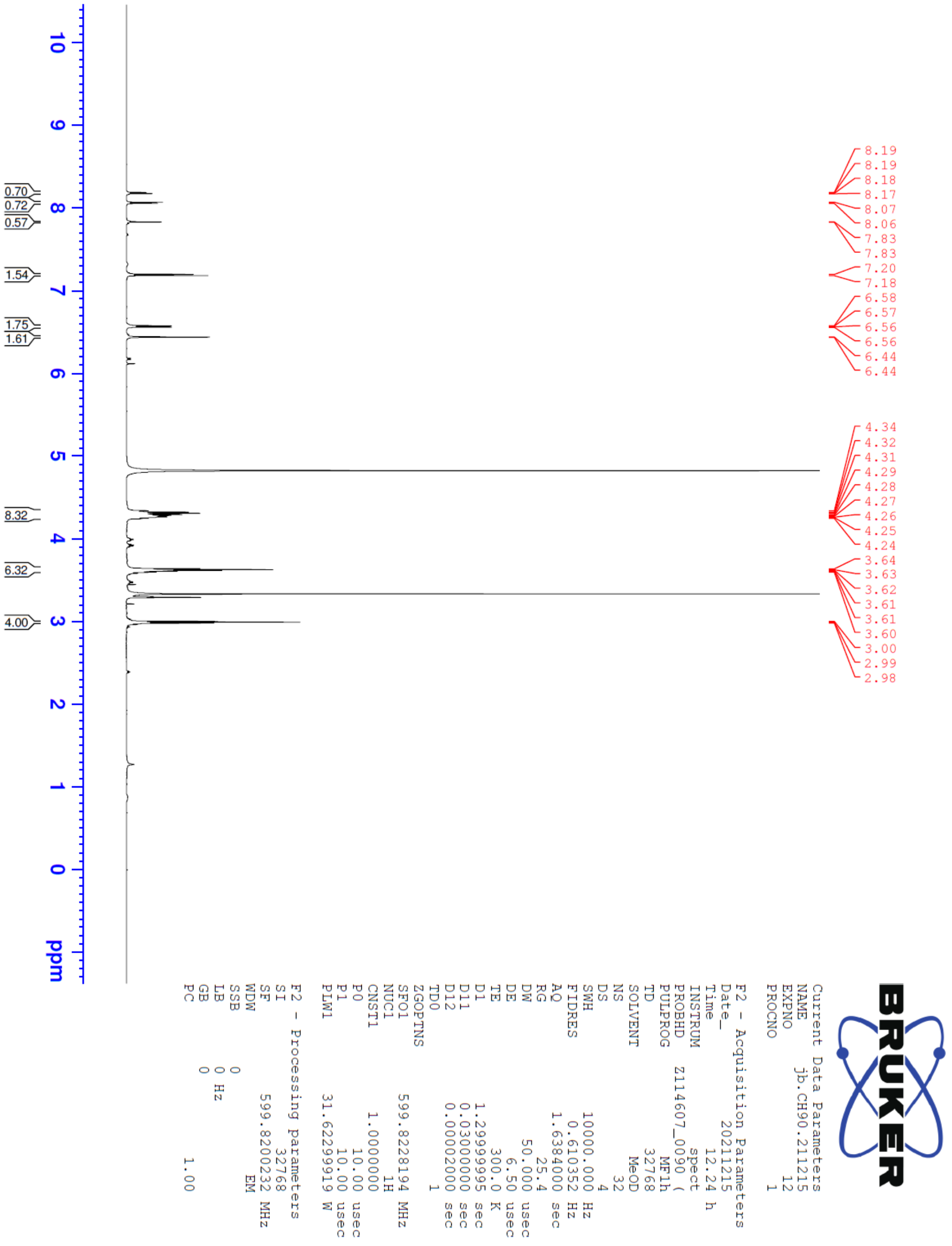

3.2. 4-Carboxy-2-(3-(3-((2-sulfoethyl)carbamoyl)azetidin-1-ium-1-ylidene)-6-(3-((2-sulfoethyl)carbamoyl)azetidin-1-yl)-3*H*-xanthen-9-yl)benzoate (3a)

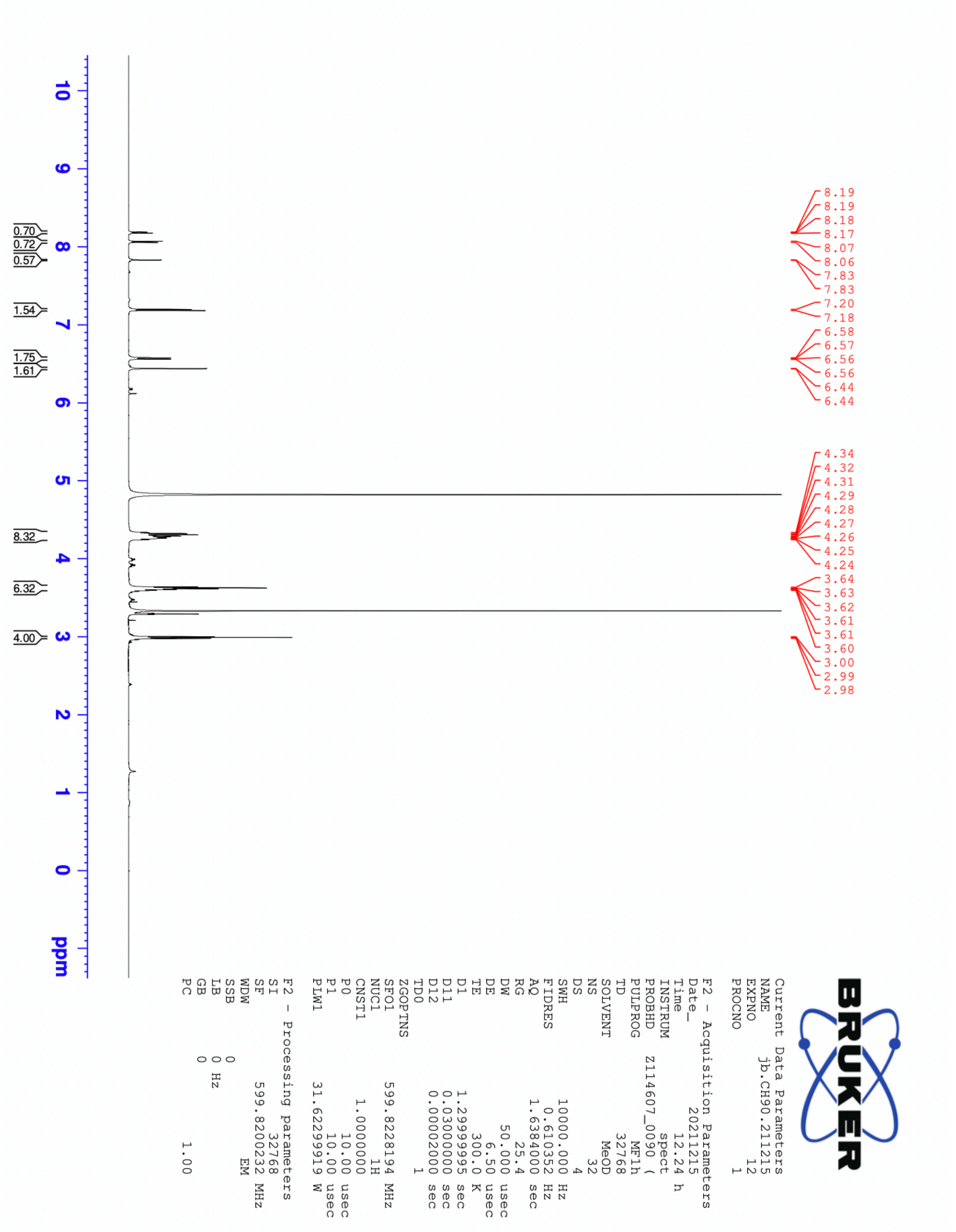

3.3. 4-Carboxy-2-(3-(3-((2-sulfoethyl)carbamoyl)azetidin-1-ium-1-ylidene)-6-(3-((2-sulfoethyl)carbamoyl)azetidin-1-yl)-3*H*-xanthen-9-yl)benzoate (3a)4-((4-(((2-Amino-9*H*-purin-6-yl)oxy)methyl)benzyl)carbamoyl)-2-(3-(3-((2-sulfoethyl)carbamoyl)azetidin-1-ium-1-ylidene)-6-(3-((2-sulfoethyl)carbamoyl)-azetidin-1-yl)-3*H*-xanthen-9-yl)benzoate (BG-Sulfo549)

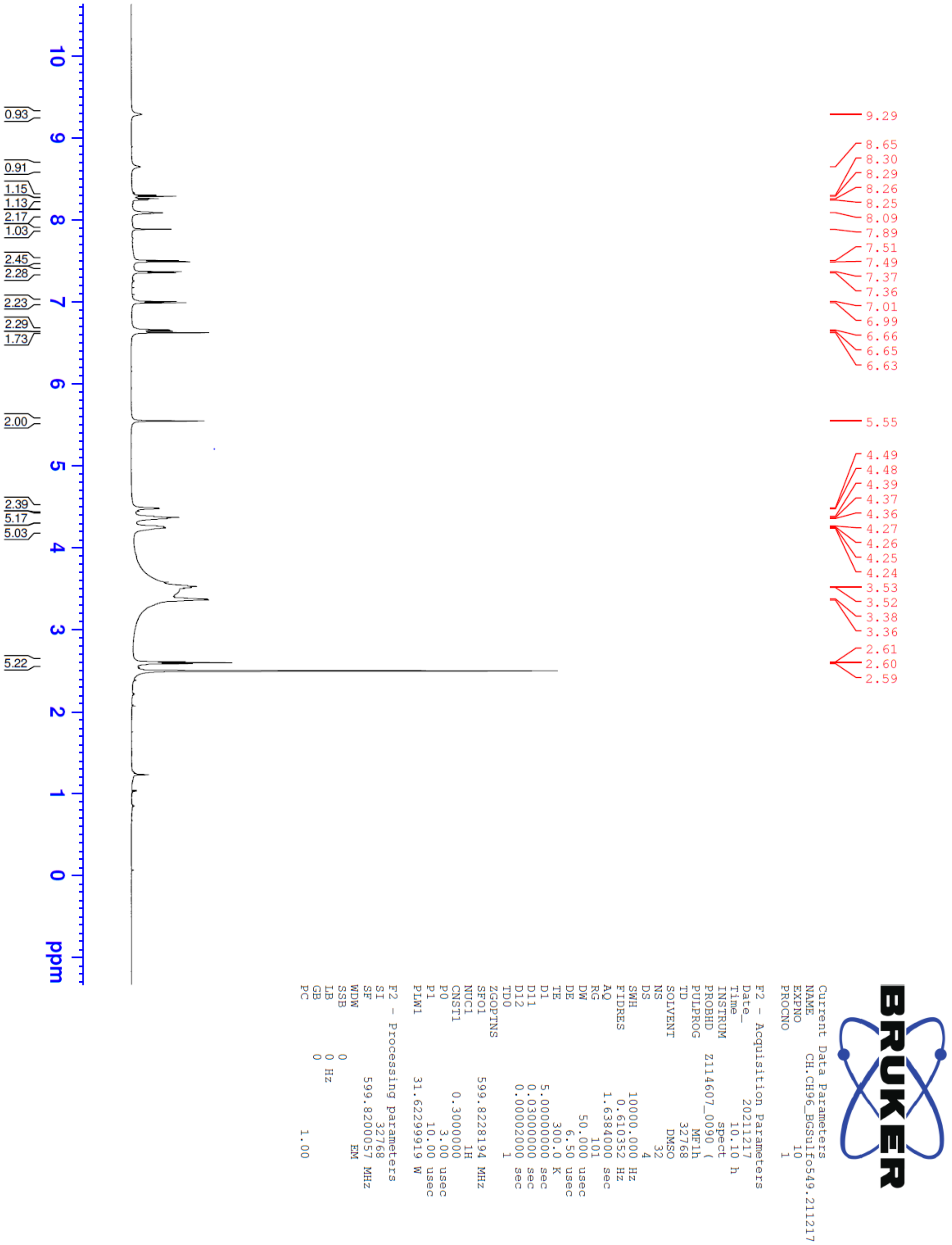

3.4. 4-((2-(2-((6-Chlorohexyl)oxy)ethoxy)ethyl)carbamoyl)-2-(3-(3-((2-sulfoethyl)-carbamoyl)azetidin-1-ium-1-ylidene)-6-(3-((2-sulfoethyl)carbamoyl)azetidin-1-yl)-3*H*-xanthen-9-yl)benzoate (CA-Sulfo549)

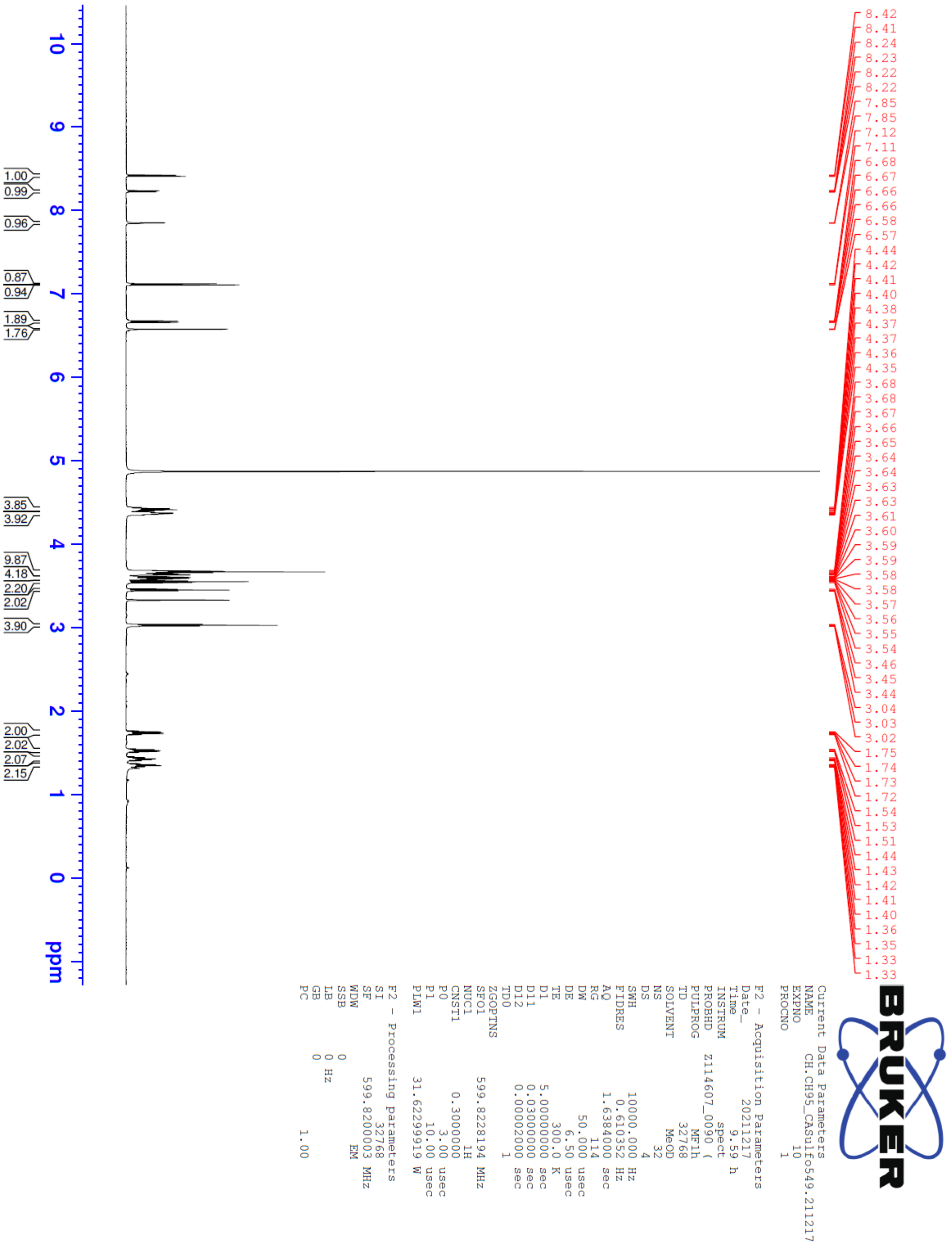

3.5. 4-(*tert*-Butoxycarbonyl)-2-(3-(3-carboxyazetidin-1-ium-1-ylidene)-7-(3-carboxy-azetidin-1-yl)-5,5-dimethyl-3,5-dihydrodibenzo[*b*,*e*]silin-10-yl)benzoate (2b)

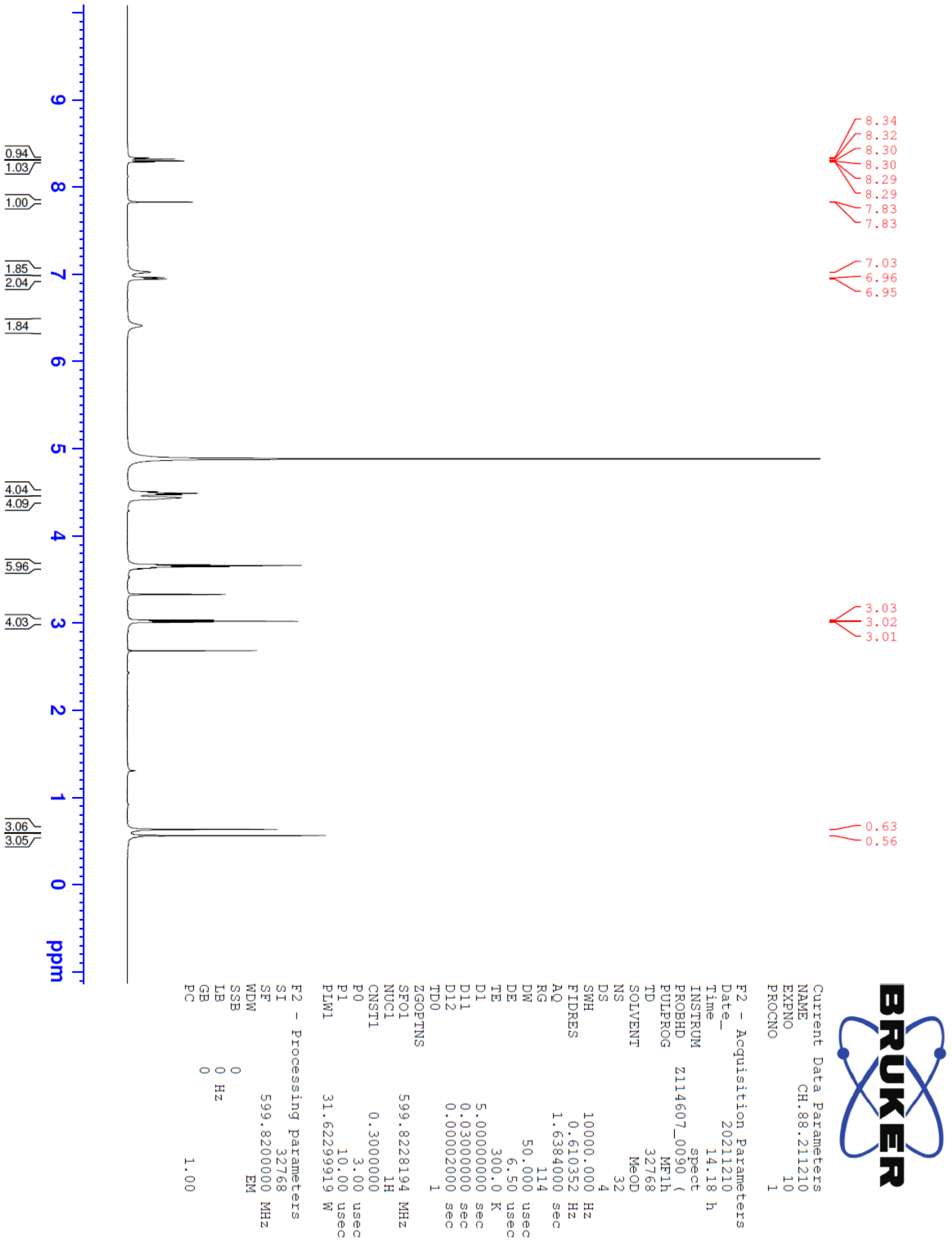

3.6. 4-Carboxy-2-(5,5-dimethyl-3-(3-((2-sulfoethyl)carbamoyl)azetidin-1-ium-1-ylidene)-7-(3-((2-sulfoethyl)carbamoyl)azetidin-1-yl)-3,5-dihydrodibenzo[*b*,*e*]silin-10-yl)benzoate (3b)

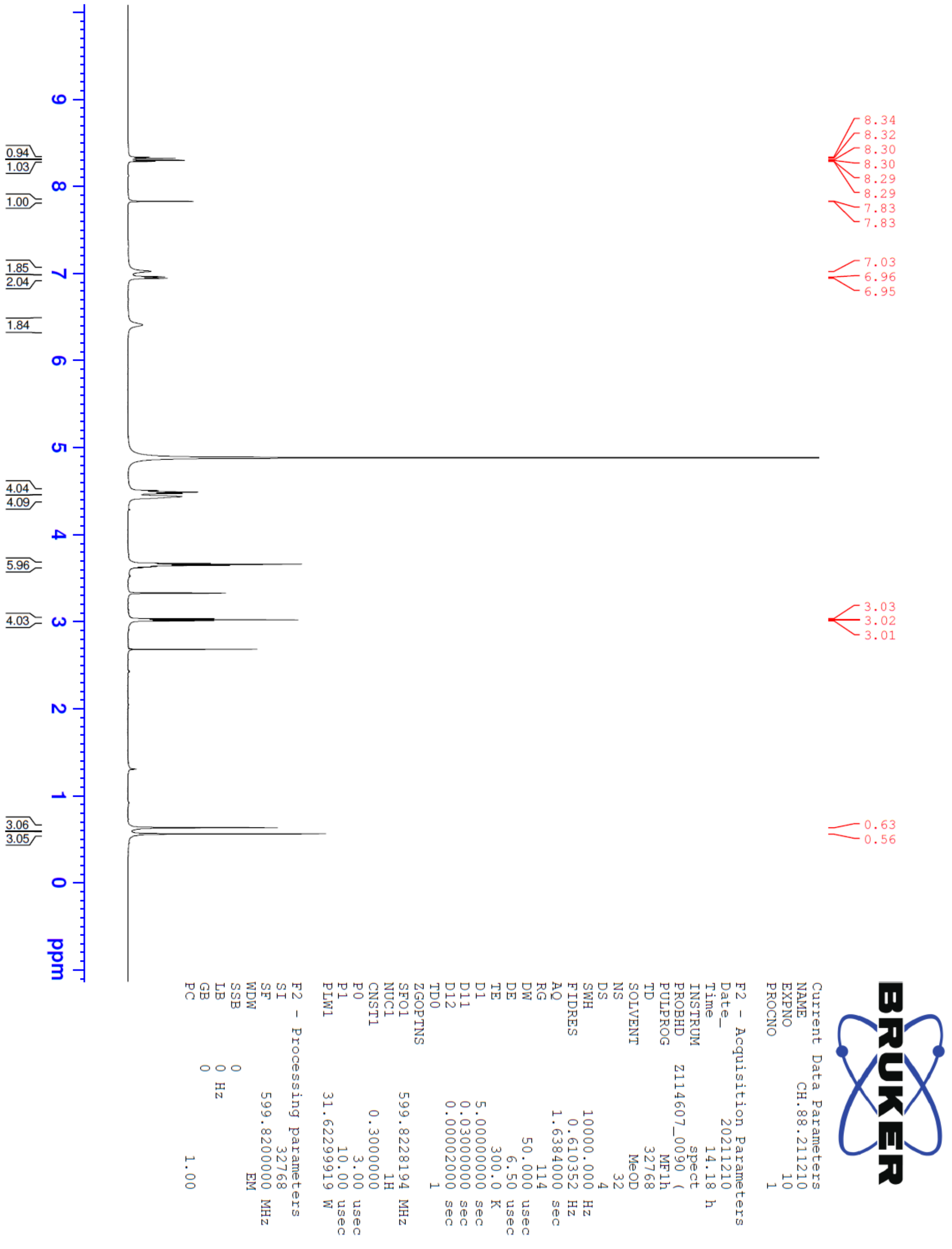

3.7. 4-((4-(2-(2-Amino-9*H*-purin-6-yl)ethyl)benzyl)carbamoyl)-2-(5,5-dimethyl-3-(3-((2-sulfoethyl)carbamoyl)azetidin-1-ium-1-ylidene)-7-(3-((2-sulfoethyl)-carbamoyl)azetidin-1-yl)-3,5-dihydrodibenzo[*b*,*e*]silin-10-yl)benzoate (BG-Sulfo646)

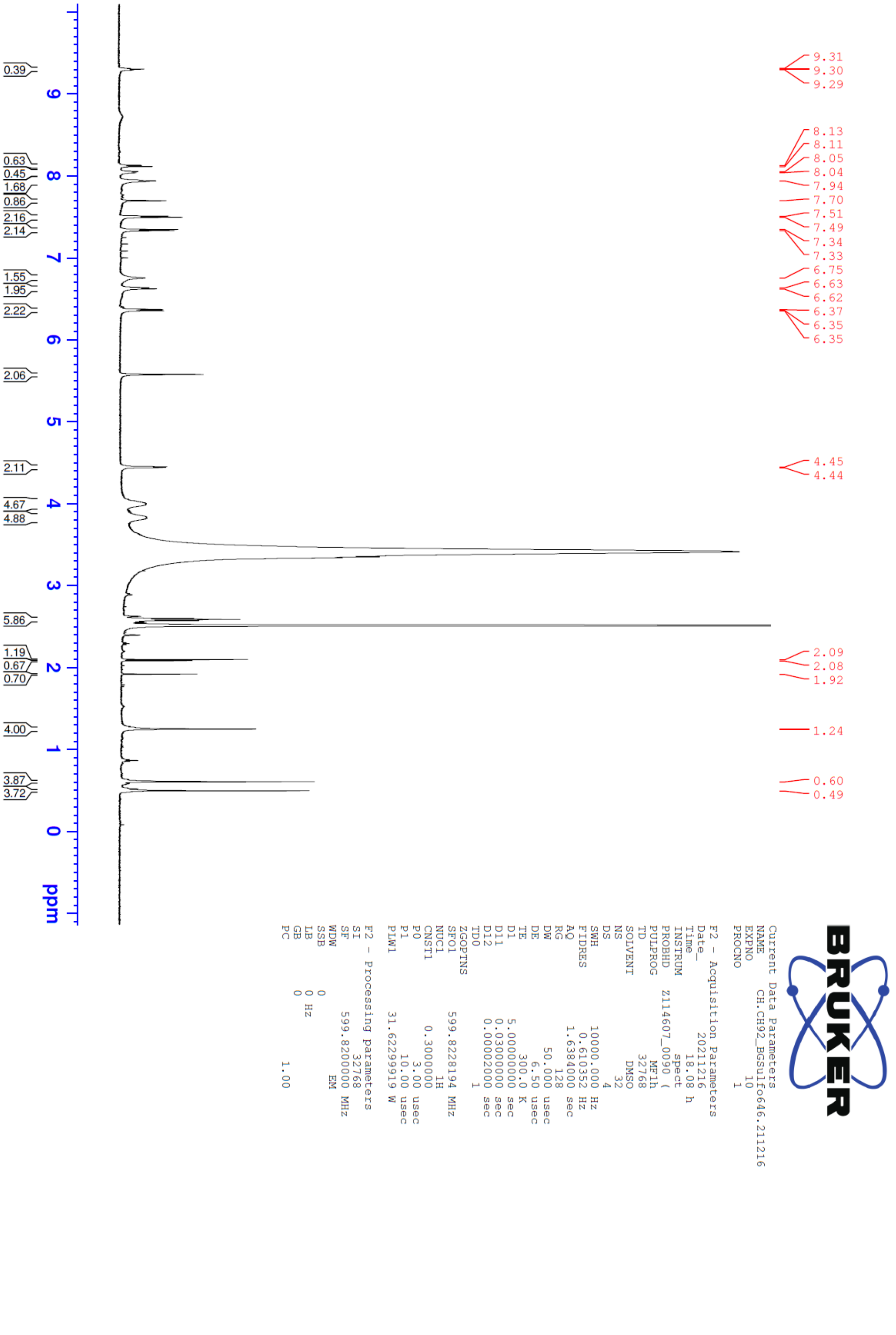

3.8. 4-((2-(2-((6-Chlorohexyl)oxy)ethoxy)ethyl)carbamoyl)-2-(5,5-dimethyl-3-(3-((2-sulfoethyl)carbamoyl)azetidin-1-ium-1-ylidene)-7-(3-((2-sulfoethyl)-carbamoyl)azetidin-1-yl)-3,5-dihydrodibenzo[*b*,*e*]silin-10-yl)benzoate (CA-Sulfo646)

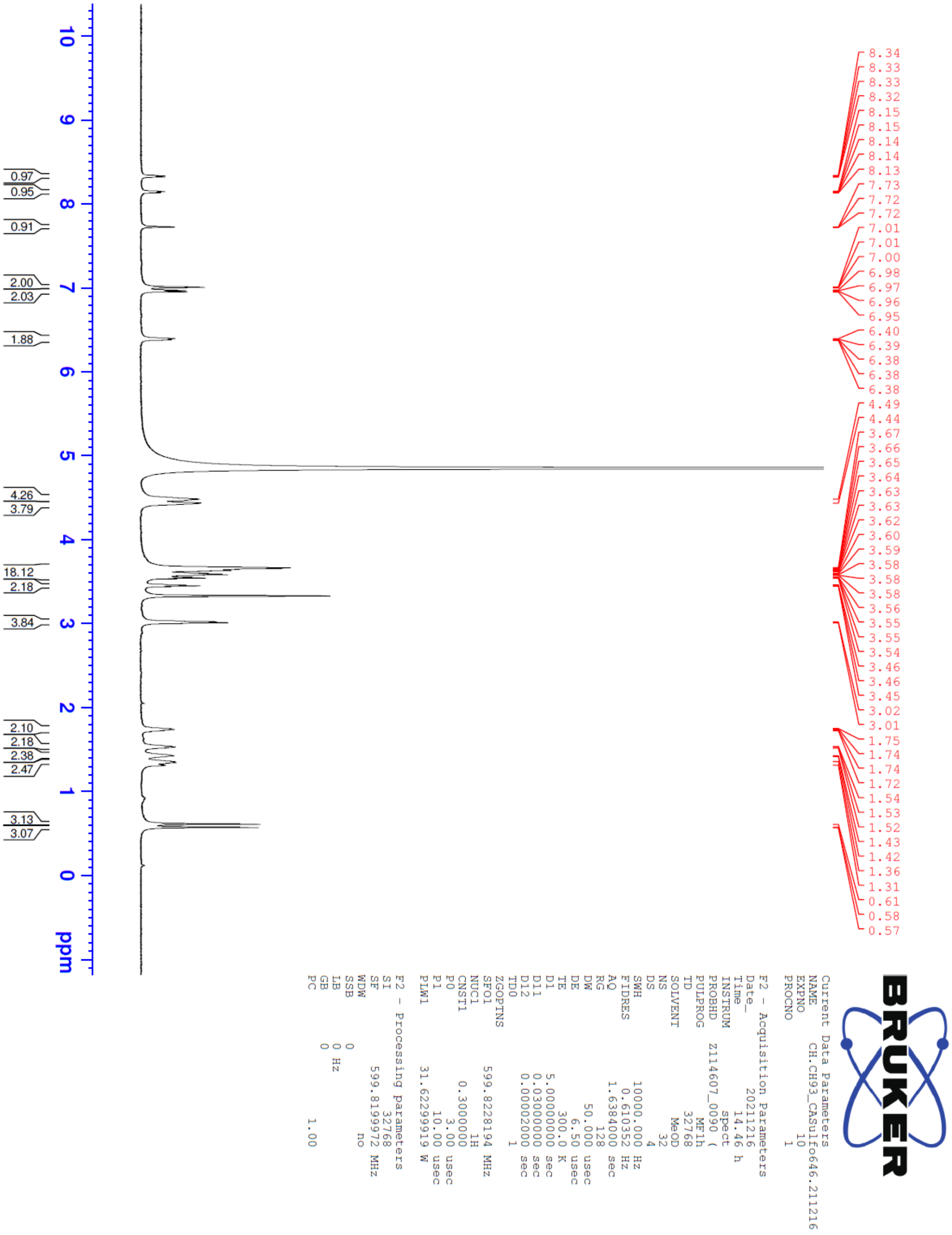

4. Protein mass spectrometry

SNAP$_f$ sequence:
MASWSHPQFEKGADDDDKVPHMDKDCEMKRTTLDSPLGKLELSGCEQGLHRIIFLGKGTSAADAVEVPAP
AAVLGGPEPLMQATAWLNAYFHQPEAIEEFPVPALHHPVFQQESFTRQVLWKLLKVVKFGEVISYSHLAA
LAGNPAATAAVKTALSGNPVPILIPCHRVVQGDLDVGGYEGGLAVKEWLLAHEGHRLGKPGLGAPGFSSI
SAHHHHHHHHHH

Strep-Tag II, Enterokinase-site, SNAPf, His-Tag

After His-tag purification, two posttranslational SNAP$_f$ constructs were observed: one with removed start codon SNAP$_f$ (2–222) and one without the N-terminal Strep-Tag II and Enterokinase-site SNAP$_f$ (22–222):

	calc.	found	Δ ppm
SNAP$_f$ (2–222)	23865.101	23865.141	1.67
SNAP$_f$ (22–222)	21618.103	21618.142	1.80
SNAP$_f$ (2–222):JF$_{549}$	24420.317	24420.357	1.65
SNAP$_f$ (22–222):JF$_{549}$	22173.319	22173.349	1.36
SNAP$_f$ (2–222):JF$_{646}$	24462.386	24462.386	0.00
SNAP$_f$ (22–222):JF$_{646}$	22215.358	22215.386	1.72
SNAP$_f$ (2–222):Sulfo549	24722.305	24722.340	1.43
SNAP$_f$ (22–222):Sulfo549	22475.307	22475.337	1.35
SNAP$_f$ (2–222):Sulfo646	24764.334	24764.387	2.16
SNAP$_f$ (22–222):Sulfo646	22517.336	22517.387	2.28

Halo sequence:
MHHHHHHHHHHENLYFQGIGTGFPFDPHYVEVLGERMHYVDVGPRDGTPVLFLHGNPTSSYVWRNIIPHV
APTHRCIAPDLIGMGKSDKPDLGYFFDDHVRFMDAFIEALGLEEVVLVIHDWGSALGFHWAKRNPERVKG
IAFMEFIRPIPTWDEWPEFARETFQAFRTTDVGRKLIIDQNVFIEGTLPMGVVRPLTEVEMDHYREPFLN
PVDREPLWRFPNELPIAGEPANIVALVEEYMDWLHQSPVPKLLFWGTPGVLIPPAEAARLAKSLPNCKAV
DIGPGLNLLQEDNPDLIGSEIARWLSTLEI

Halo, His-Tag

	calc.	found	Δ ppm
Halo	35465.901	35465.985	2.37
Halo:JF$_{549}$	36089.251	36089.293	1.16
Halo:JF$_{646}$	36131.230	36131.297	1.85
Halo:Sulfo549	36391.188	36391.291	2.83
Halo:Sulfo646	36433.217	36433.317	2.74

5. Protein constructs

SNAP–TM–Halo sequence:

METDTLLLWVLLLWVPGSTGDYPYDVPDYAGAQPARSMDKDCEMKRTTLDSPLGKLELSGCEQGLHEIIF
LGKGTSAADAVEVPAPAAVLGGPEPLMQATAWLNAYFHQPEAIEEFPVPALHHPVFQQESFTRQVLWKLL
KVVKFGEVISYSHLAALAGNPAATAAVKTALSGNPVPILIPCHRVVQGDLDVGGYEGGLAVKEWLLAHEG
HRLGKPGLGGRLEVLFQGVDEQKLISEEDLNAVGQDTQEVIVVPHSLPFKVVVISAILALVVLTIISLII
LIMLWQKKPRGAQPARSGSEIGTGFPFDPHYVEVLGERMHYVDVGPRDGTPVLFLHGNPTSSYVWRNIIP
HVAPTHRCIAPDLIGMGKSDKPDLGYFFDDHVRFMDAFIEALGLEEVVLVIHDWGSALGFHWAKRNPERV
KGIAFMEFIRPIPTWDEWPEFARETFQAFRTTDVGRKLIIDQNVFIEGTLPMGVVRPLTEVEMDHYREPF
LNPVDREPLWRFPNELPIAGEPANIVALVEEYMDWLHQSPVPKLLFWGTPGVLIPPAEAARLAKSLPNCK
AVDIGPGLNLLQEDNPDLIGSEIARWLSTLEISGVD*

IgK signal peptide, HA-tag, SNAP, myc, transmembrane domain, Halo-Tag

Halo–TM–SNAP sequence:

METDTLLLWVLLLWVPGSTGDYPYDVPDYAGAQPARSGSEIGTGFPFDPHYVEVLGERMHYVDVGPRDGT
PVLFLHGNPTSSYVWRNIIPHVAPTHRCIAPDLIGMGKSDKPDLGYFFDDHVRFMDAFIEALGLEEVVLV
IHDWGSALGFHWAKRNPERVKGIAFMEFIRPIPTWDEWPEFARETFQAFRTTDVGRKLIIDQNVFIEGTL
PMGVVRPLTEVEMDHYREPFLNPVDREPLWRFPNELPIAGEPANIVALVEEYMDWLHQSPVPKLLFWGTP
GVLIPPAEAARLAKSLPNCKAVDIGPGLNLLQEDNPDLIGSEIARWLSTLEISGVDEQKLISEEDLNAVG
QDTQEVIVVPHSLPFKVVVISAILALVVLTIISLIILIMLWQKKPRGAQPARSMDKDCEMKRTTLDSPLG
KLELSGCEQGLHEIIFLGKGTSAADAVEVPAPAAVLGGPEPLMQATAWLNAYFHQPEAIEEFPVPALHHP
VFQQESFTRQVLWKLLKVVKFGEVISYSHLAALAGNPAATAAVKTALSGNPVPILIPCHRVVQGDLDVGG
YEGGLAVKEWLLAHEGHRLGKPGLGGRLEVLFQGVD*

IgK signal peptide, HA-tag, Halo-Tag, myc, transmembrane domain, SNAP

Halo-GLP1R sequence:

MALPVTALLLPLALLLHAARPAAASGIDYKDDDDKAGIDAIGSEIGTGFPFDPHYVEVLGERMHYVDVGP
RDGTPVLFLHGNPTSSYVWRNIIPHVAPTHRCIAPDLIGMGKSDKPDLGYFFDDHVRFMDAFIEALGLEE
VVLVIHDWGSALGFHWAKRNPERVKGIAFMEFIRPIPTWDEWPEFARETFQAFRTTDVGRKLIIDQNVFI
EGTLPMGVVRPLTEVEMDHYREPFLNPVDREPLWRFPNELPIAGEPANIVALVEEYMDWLHQSPVPKLLF
WGTPGVLIPPAEAARLAKSLPNCKAVDIGPGLNLLQEDNPDLIGSEIARWLSTLEISGDIRPQGATVSLW
ETVQKWREYRRQCQRSLTEDPPPATDLFCNRTFDEYACWPDGEPGSFVNVSCPWYLPWASSVPQGHVYRF
CTAEGLWLQKDNSSLPWRDLSECEESKRGERSSPEEQLLFLYIIYTVGYALSFSALVIASAILLGFRHLH
CTRNYIHLNLFASFILRALSVFIKDAALKWMYSTAAQQHQWDGLLSYQDSLSCRLVFLLMQYCVAANYYW
LLVEGVYLYTLLAFSVLSEQWIFRLYVSIGWGVPLLFVVPWGIVKYLYEDEGCWTRNSNMNYWLIIRLPI
LFAIGVNFLIFVRVICIVVSKLKANLMCKTDIKCRLAKSTLTLIPLLGTHEVIFAFVMDEHARGTLRFIK
LFTELSFTSFQGLMVAILYCFVNNEVQLEFRKSWERWRLEHLHIQRDSSMKPLKCPTSSLSSGATAGSSM
YTATCQASCS*

CD8 signal peptide, FLAG-tag, Halo-tag, GLP1R

6. Supplemental Figures

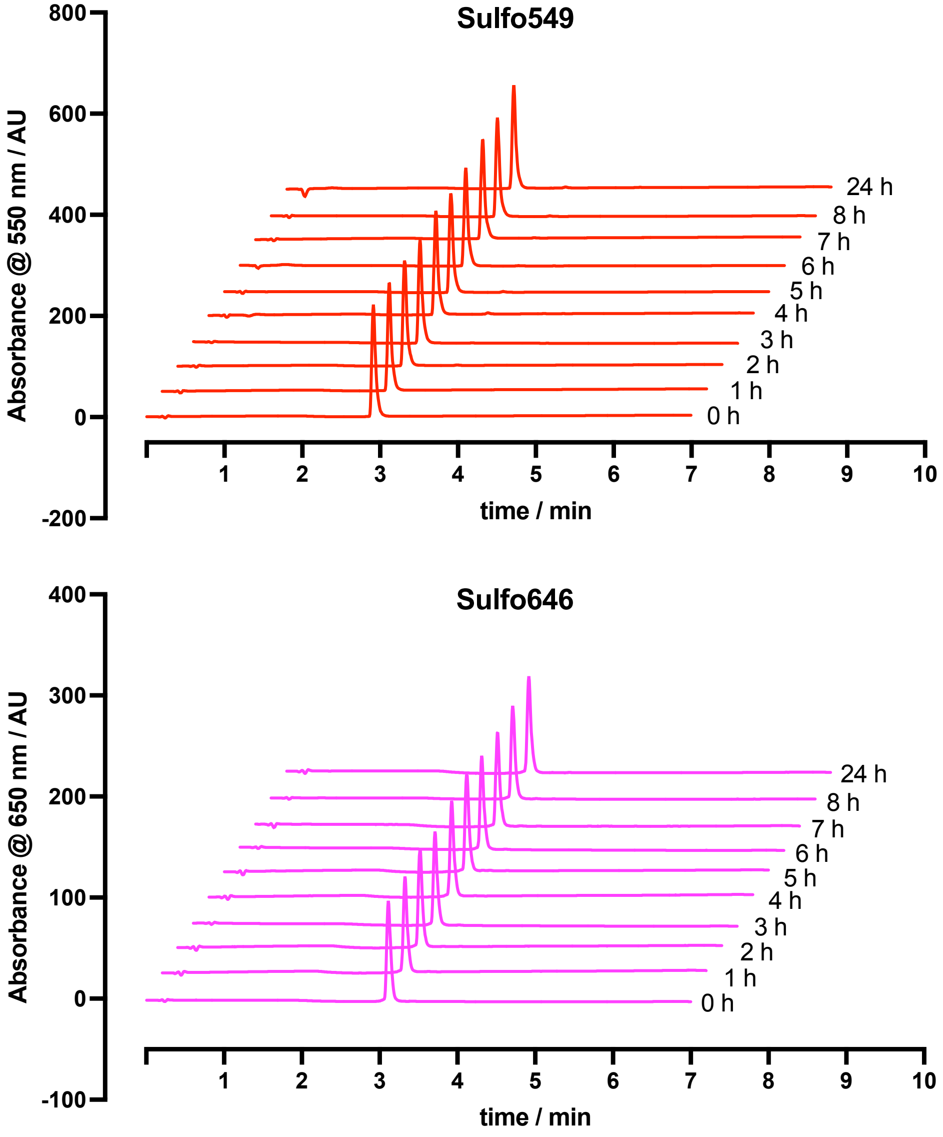

Supplemental Figure S1: Stability of Sulfo549 and Sulfo646 in PBS measured by LCMS over 24 hours.

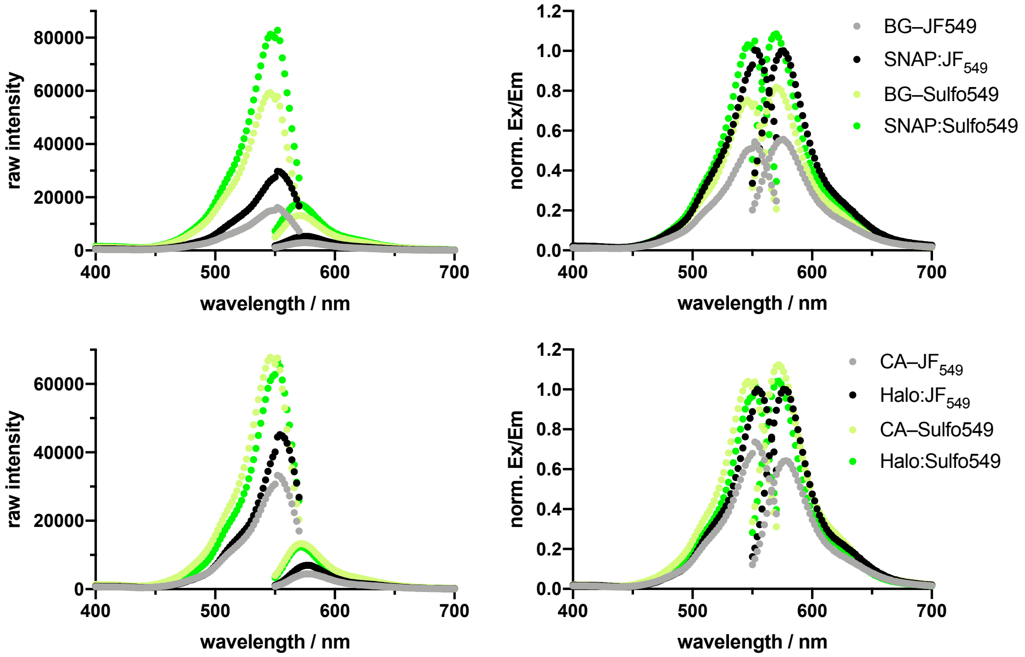

Supplemental Figure S2: Excitation and emission spectra of bound and unbound 549 probes with raw intensity (left) and normalized to JF dye excitation and emission (right, cf. Figure 2).

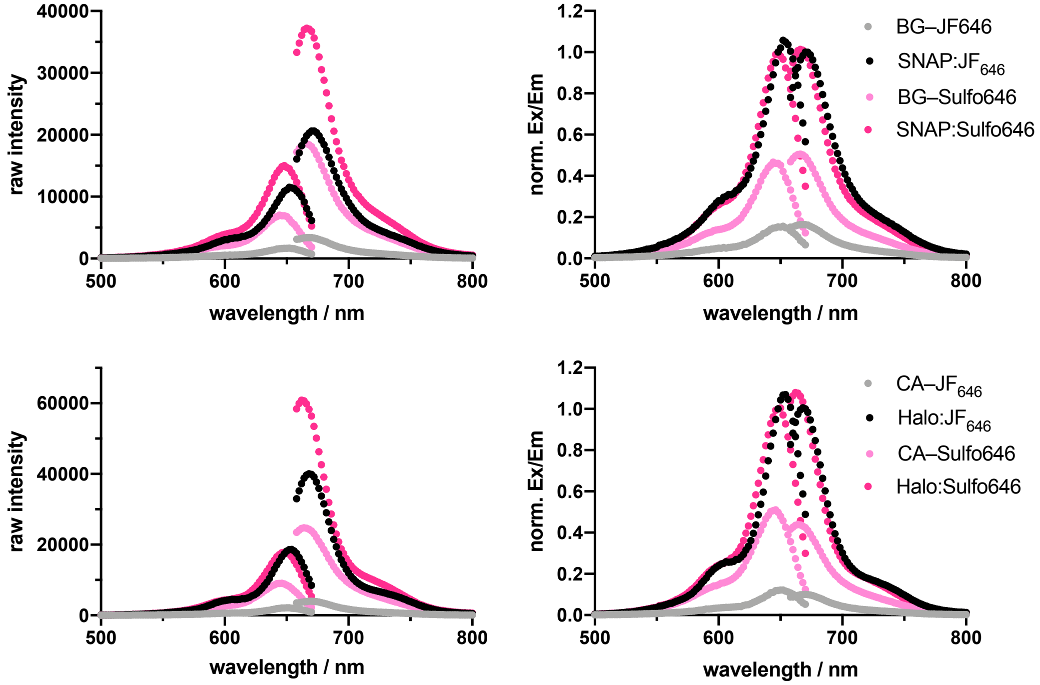

Supplemental Figure S3: Excitation and emission spectra of bound and unbound 646 probes with raw intensity (left) and normalized to JF dye excitation and emission (right, cf. Figure 2).

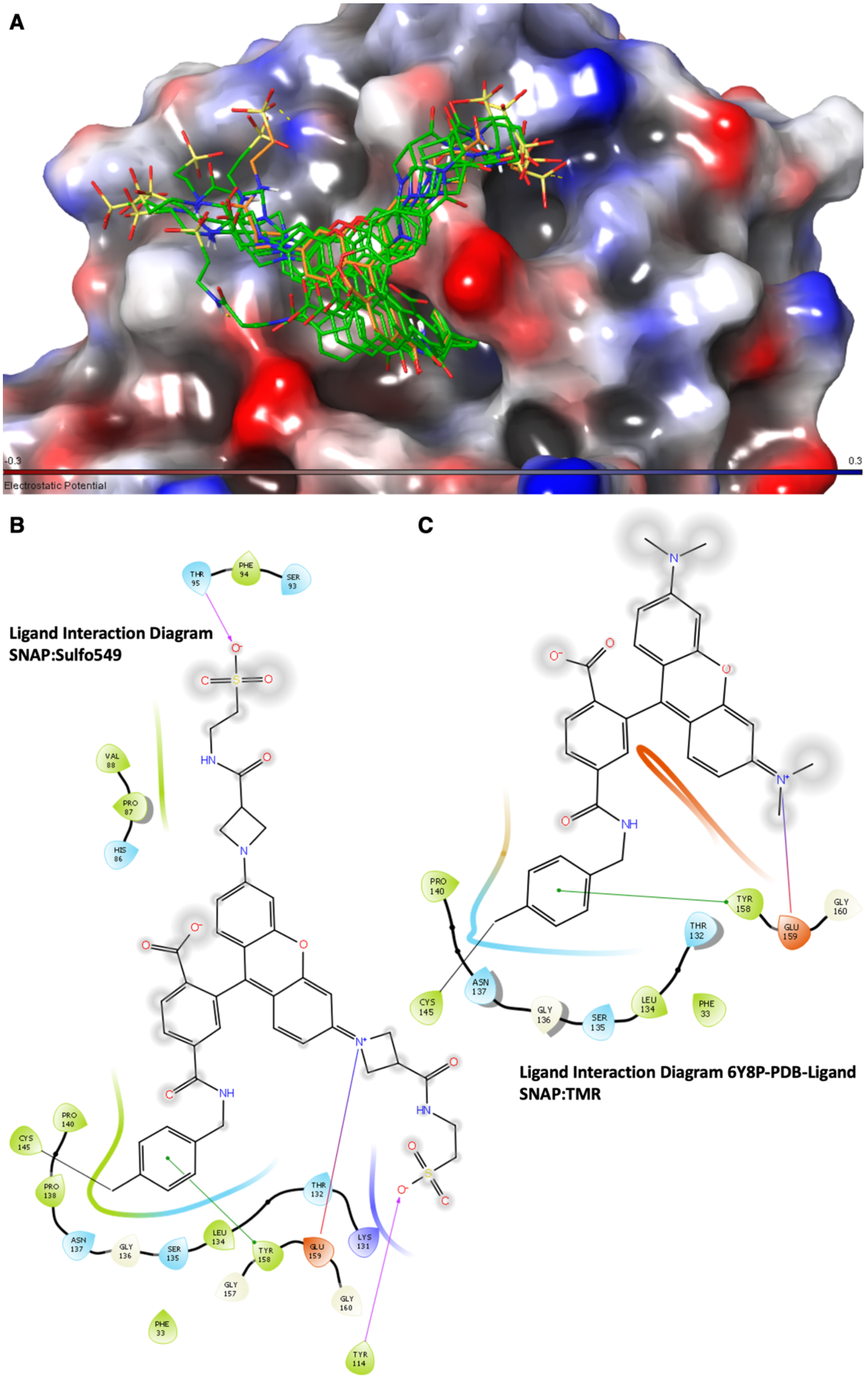
A
-0.3
0.3
Electrostatic Potential
B
Ligand Interaction Diagram
SNAP:Sulfo549
C
Ligand Interaction Diagram 6Y8P-PDB-Ligand
SNAP:TMR

Supplemental Figure S4: Molecular docking of Sulfo549. A) Electrostatic potential surface show 8 out of 10 poses with good overlap. Pose 1: orange C atoms; hydrogen bonds to the sulfonates from Thr95 and Tyr114 are dashed yellow. **B)** Several new interactions predicted between Sulfo549 ligand with the SNAP protein surface that TMR could not make and is not observed in the X-ray structure pdb: 6y8p.

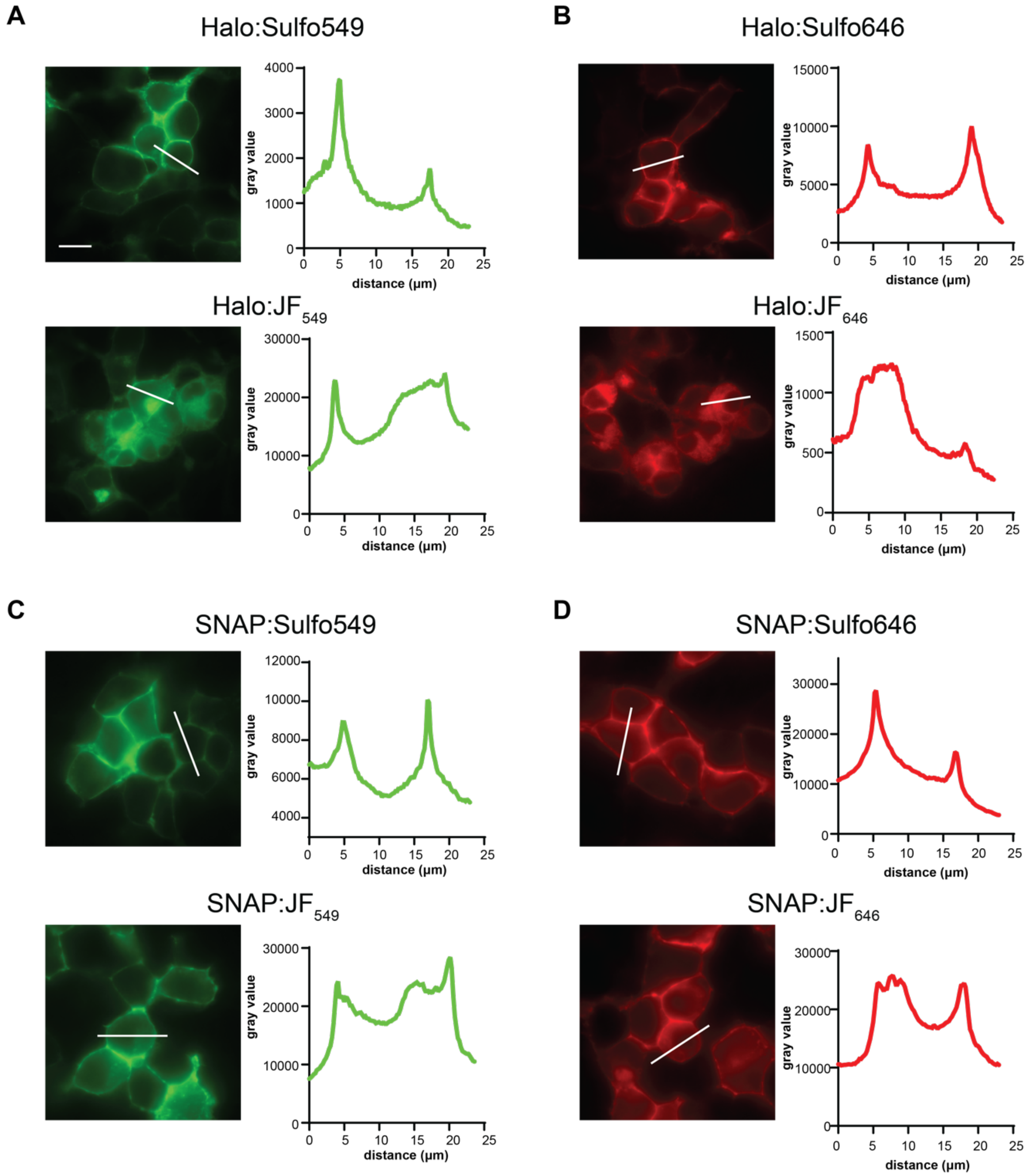

Supplemental Figure S5: HEK293 cells transfected with SNAP- or Halo-β2AR and labelled with JaneliaFluor or Sulfo Dyes. A) Halo-β2AR transfected cells stained with CA-Sulfo549 vs. CA-JF_{549}. **B)** Halo-β2AR transfected cells stained with CA-Sulfo646 vs. CA-JF_{646}. **C)** SNAP-β2AR transfected cells stained with BG-Sulfo549 vs. BG-JF_{549}. **D)** SNAP-β2AR transfected cells stained with BG-Sulfo646 vs. BG-JF_{646}. Scale bar = 10 µm.

7. Supplemental Schemes

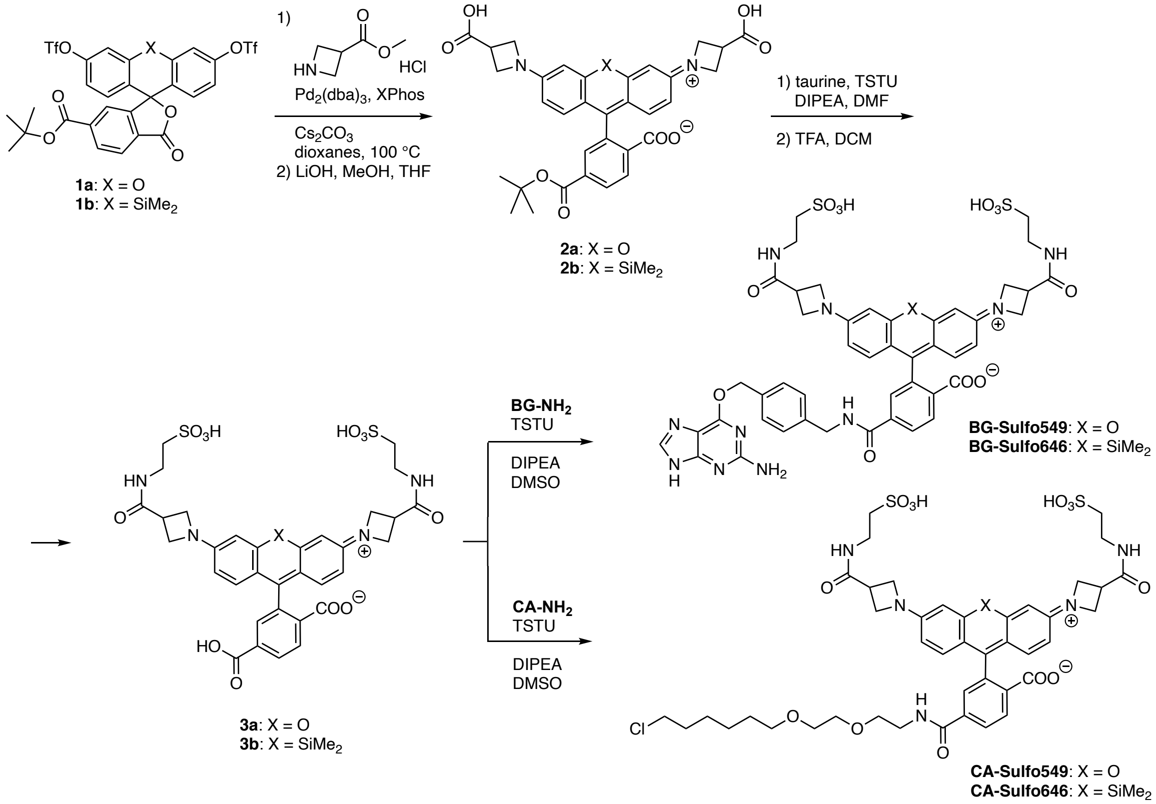

Supplemental Scheme 1: Synthesis of Sulfo dyes.

8. References

[1] A. Acosta-Ruiz, V. A. Gutzeit, M. J. Skelly, S. Meadows, J. Lee, P. Parekh, A. G. Orr, C. Liston, K. E. Pleil, J. Broichhagen, J. Levitz, *Neuron* **2020**, *105*, 446-463.
